# Monocyte-derived alveolar macrophages are key drivers of smoke-induced lung inflammation and tissue remodeling

**DOI:** 10.3389/fimmu.2024.1325090

**Published:** 2024-01-29

**Authors:** Christian T. Wohnhaas, Kevin Baßler, Carolin K. Watson, Yang Shen, Germán G. Leparc, Cornelia Tilp, Fabian Heinemann, David Kind, Birgit Stierstorfer, Denis Delić, Thomas Brunner, Florian Gantner, Joachim L. Schultze, Coralie Viollet, Patrick Baum

**Affiliations:** ^1^ Translational Medicine & Clinical Pharmacology, Boehringer Ingelheim Pharma GmbH & Co. KG, Biberach, Germany; ^2^ Genomics and Immunoregulation, Life & Medical Sciences (LIMES) Institute, University of Bonn, Bonn, Germany; ^3^ Immunology & Respiratory Research, Boehringer Ingelheim Pharma GmbH & Co. KG, Biberach, Germany; ^4^ Global Computational Biology and Digital Sciences, Boehringer Ingelheim Pharma GmbH & Co. KG, Biberach, Germany; ^5^ Drug Discovery Sciences, Boehringer Ingelheim Pharma GmbH & Co. KG, Biberach, Germany; ^6^ Fifth Department of Medicine (Nephrology/Endocrinology/Rheumatology), University Medical Centre Mannheim, University of Heidelberg, Mannheim, Germany; ^7^ Department of Biology, University of Konstanz, Konstanz, Germany; ^8^ Translational Medicine & Clinical Pharmacology, C. H. Boehringer Sohn AG & Co. KG, Biberach, Germany; ^9^ Systems Medicine, German Center for Neurodegenerative Diseases (DZNE), Bonn, Germany; ^10^ PRECISE Platform for Single Cell Genomics and Epigenomics, German Center for Neurodegenerative Diseases (DZNE) and University of Bonn, Bonn, Germany

**Keywords:** monocyte-derived alveolar macrophages, single-cell RNA-sequencing, COPD, lung inflammation, IRAK4 inhibitor, neutrophils, smoking, tissue remodeling

## Abstract

Smoking is a leading risk factor of chronic obstructive pulmonary disease (COPD), that is characterized by chronic lung inflammation, tissue remodeling and emphysema. Although inflammation is critical to COPD pathogenesis, the cellular and molecular basis underlying smoking-induced lung inflammation and pathology remains unclear. Using murine smoke models and single-cell RNA-sequencing, we show that smoking establishes a self-amplifying inflammatory loop characterized by an influx of molecularly heterogeneous neutrophil subsets and excessive recruitment of monocyte-derived alveolar macrophages (MoAM). In contrast to tissue-resident AM, MoAM are absent in homeostasis and characterized by a pro-inflammatory gene signature. Moreover, MoAM represent 46% of AM in emphysematous mice and express markers causally linked to emphysema. We also demonstrate the presence of pro-inflammatory and tissue remodeling associated MoAM orthologs in humans that are significantly increased in emphysematous COPD patients. Inhibition of the IRAK4 kinase depletes a rare inflammatory neutrophil subset, diminishes MoAM recruitment, and alleviates inflammation in the lung of cigarette smoke-exposed mice. This study extends our understanding of the molecular signaling circuits and cellular dynamics in smoking-induced lung inflammation and pathology, highlights the functional consequence of monocyte and neutrophil recruitment, identifies MoAM as key drivers of the inflammatory process, and supports their contribution to pathological tissue remodeling.

## Graphical Abstract

The graphical abstract was created with Biorender.com.

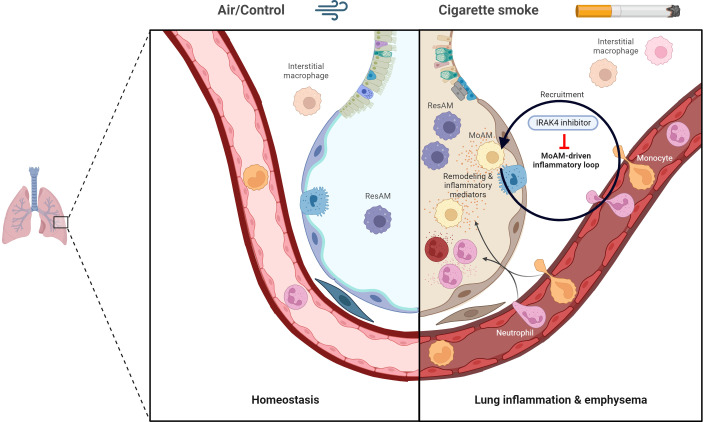

## 1 Introduction

Chronic obstructive pulmonary disease (COPD) is a progressive inflammatory disorder with a global prevalence of 10.3% and an estimated 392 million patients worldwide (1, 2). Chronic inflammation of the peripheral airways and lung parenchyma are characteristics of the disease, which holds also true for irreversible airflow limitation that is related to alterations of the lung structure (3–5). These structural alterations include small airway remodeling and obstruction owing to small airway fibrosis and emphysema, that is characterized by a loss of alveolar septa and enlarged alveoli (3, 5).

Cigarette smoking is the major risk factor for COPD in the Western world (4) and known to induce tissue injury in the lung either directly by oxidative stress or indirectly by inflammation (6). Inflammation is key to COPD pathogenesis and initiated by pattern recognition receptors (PRRs), including Toll-like receptors (TLRs), that are activated through chronically inhaled irritants or indirectly by damage-associated molecular patterns that are released upon cell damage and trigger an innate immune response (5, 7–9). In the further course of the disease, COPD is characterized by abnormal levels of inflammatory cytokines, such as the inflammatory master regulator IL-1β, and chemoattractants that trigger the recruitment of immune cells from the bloodstream into the lung (10–12). In accordance with that, various immune cells of the innate and adaptive immunity are eventually involved in the inflammatory response (3, 11), arguing for a highly diverse immune cell population and complex cellular interplay in the smoke-induced inflammatory environment. Among these cell populations, neutrophils and macrophages seem to play an important role as their increase was shown to be related to disease severity (3, 11).

The lung harbors different subsets of macrophages, including interstitial macrophages that are present in the lung interstitium between blood vessels and alveoli whereas alveolar macrophages (AM) are found in the alveolar space and airspace (13, 14). Tissue-resident AM (ResAM) originate from fetal monocytes and are able to self-maintain under homeostasis in mice (13, 15, 16). In contrast, recent studies demonstrated that bone marrow-derived monocytes are recruited into the lung and contribute to the AM pool during inflammation (16, 17). Following the same concept, monocyte recruitment into the lung in response to chemoattractants such as CCL2 is thought to contribute to the increase of macrophages in sputum of COPD patients (11, 18). These recruited monocyte-derived alveolar macrophages (MoAM), but not ResAM, were recently identified as the key drivers of pulmonary fibrosis in murine models (16, 19). In contrast, the cellular and molecular basis, including the role of MoAM, underlying smoking-related lung inflammation and its progression from an acute to the chronic pathologically relevant state remain poorly understood. This is also reflected by the lack of disease-modifying (4, 20) and preventive therapies for COPD.

Single-cell genomics technologies, particularly single-cell RNA-sequencing (scRNA-seq), now allows a detailed characterization of such complex cell communities and their molecular mechanisms. Here, we used scRNA-seq to increase the understanding of the cellular dynamics and molecular profiles of resident and recruited immune cells in cigarette smoke (CS)-induced lung inflammation with special emphasis on AM and neutrophils in the alveolar space using murine smoke models that were previously shown to reflect key features of COPD including airway inflammation and pulmonary emphysema (21, 22). In order to differentiate early from chronic smoke-induced effects, which is important to understand the molecular and cellular mechanisms driving the progression to the irreversible stage of pathological tissue remodeling, we first characterized the acute (4 days) state of smoke-induced lung inflammation and then focused our analysis on the sub-chronic (3 weeks) and chronic (12 weeks) stages. This integrative approach revealed that smoking establishes a self-amplifying inflammatory loop that is characterized by excessive recruitment of pro-inflammatory MoAM. These recruited MoAM, but not ResAM, were identified as key drivers of the inflammatory response. Furthermore, the massive increase of MoAM in emphysematous mice and expression of markers causally linked to emphysema strongly supports their direct involvement in pathological tissue remodeling. This is corroborated by a significant increase of human MoAM orthologs in emphysematous COPD patients, which illustrates the relevance and translatability of our findings to human disease. Additionally, we highlight different molecular subsets of neutrophils in smoke-exposed animals that were identified as important inflammatory amplifiers. Finally, we provide first proof-of-principle evidence that pharmacological inhibition of interleukin-1 receptor-associated kinase 4 (IRAK4), a central signaling node located down-stream of TLR and IL-1 receptor family members (23), reduces neutrophil and MoAM recruitment and diminishes smoke-induced inflammation.

## 2 Materials and methods

### 2.1 Animals

Eight week old female C57BL/6J mice weighing 18-23 g (Charles River Laboratories, Sulzfeld, Germany) were used for all experiments. Mice were housed in groups in individually ventilated cages (IVC) under SPF conditions within Boehringer Ingelheim’s animal facility under a 12-hour light/dark cycle. They had free access to water and food throughout their time in the IVCs.

### 2.2 Cigarette smoke exposure

**Figure 1:**
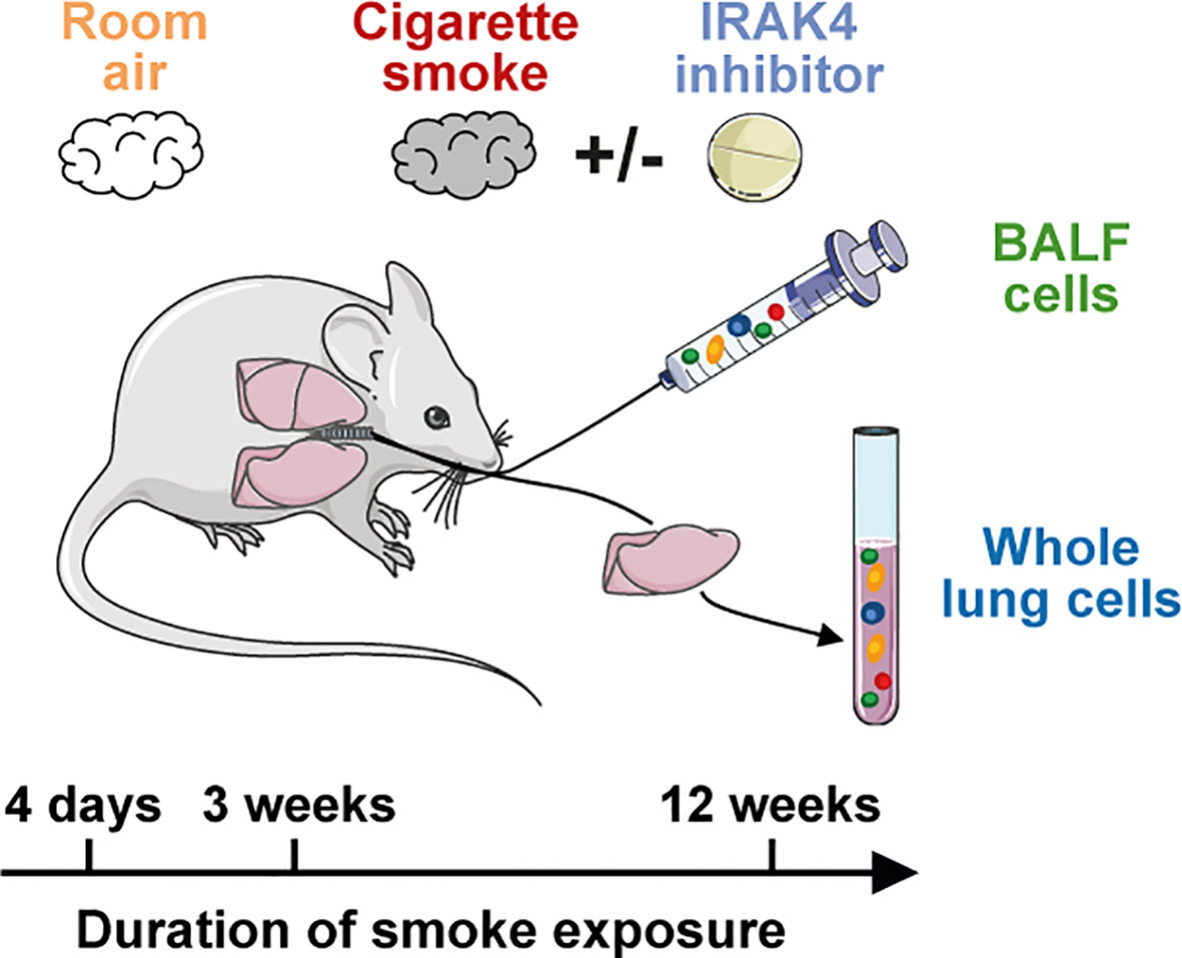
Murine smoke model. Scheme of the murine smoke model employed in the study. Mice were exposed daily to whole body mainstream cigarette smoke for 4 days (acute), 3 weeks (sub-chronic) and 12 weeks (chronic). Control animals were exposed to room air and BALF and whole lung (acute model only) immune cells were investigated by scRNA-seq. The effect of an IRAK4 inhibitor as a potential anti-inflammatory treatment was investigated by the acute and sub-chronic models.

Mice were either exposed to room air or to mainstream cigarette smoke (Roth-Händle without filters, 10 mg tar, 1.0 mg nicotine, 6 mg carbon monoxide, Reemtsma Cigarettenfabriken GmbH, Hamburg, Germany; **Figure 1**) using a semi-automatic cigarette lighter and smoke generator that was equipped with an electronic timer to regulate CS exposure (Boehringer Ingelheim Pharma GmbH & Co. KG, Biberach, Germany). Mice were seated unrestrained in a cylindrical, custom-made 32 L Perspex box which was heated to 27°C during the CS/room air exposure. Animals did not have access to food or water during CS exposure.

For the acute CS model, whole body smoke exposure was performed in the morning on four consecutive days according to the following scheme: six cigarettes on both day 1 and 2, eight cigarettes on day 3 and ten cigarettes on day 4. Mice were exposed to smoke for 16 min per cigarette, followed by a 8 min room air exposure (24 min smoking/room air cycle). At the end of the smoking/room air cycle of every other cigarette, mice were exposed to room air for 24 min. For example, on Day 1, the following durations apply: two consecutive 24 min smoking/room air cycles, room air for 24 min, 2x 24 min smoking/room air cycle, room air for 24 min, 2x 24 min smoking/room air cycle, room air for 24 min. Mice of the control group were exposed to room air instead of cigarette smoke according to the same scheme. On day 5, the animals were euthanized by i.p. injection of pentobarbital sodium (400 mg/kg) and analyzed 18 hours after the last CS exposure.

Sub-chronic and chronic smoke exposure were investigated in two independent experiments using the same apparatus as described for the acute CS model. In order to investigate the effect of sub-chronic smoke exposure, mice were exposed daily to six cigarettes in the morning for three consecutive weeks, seven days a week. For the chronic CS model, the animals were exposed daily to CS for twelve consecutive weeks, seven days a week. CS exposure was performed twice daily to four cigarettes, once early in the morning and secondly in the late afternoon with a recovery phase of 6.5 h, on five days a week. At the weekend, CS exposure was performed once daily in the morning with four cigarettes. Again, mice were exposed to room air every other cigarette as performed for the acute smoke model, while control animals were exposed to room air. Animals were sacrificed 18 h after the last smoke exposure as described for the acute CS model.

### 2.3 IRAK4 inhibitor treatment

IRAK4 inhibitor was dissolved in 0.5% Natrosol^®^/0.015% Tween^®^ 80 and a dose of 50 mg/kg was finally administered p.o. with an application volume of 10 mL/kg. Administration of the compound was performed daily 1 h prior to and 7 h post start of smoke exposure. Control animals were sham treated and received the solvent only.

### 2.4 Cell isolation from bronchoalveolar lavage fluid

Tracheas of the euthanized animals were cannuled for bronchoalveolar lavage and lungs were flushed twice using 0.8 mL of Hanks’ Balanced Salt solution. Neutrophil and macrophage counts in BALF were measured by a Sysmex XT1800 iVet analyzer (Sysmex Europe GmbH, Norderstedt, Germany). Cells were subsequently prepared for scRNA-seq analysis and stored on ice throughout the procedure. First, cells were pelleted, the supernatant was removed, and the cell pellet again resuspended in 500 µL of HBSS/BSA/EDTA buffer (0.04% BSA and 2 mM EDTA in HBSS). Then, cell pools were prepared for subsequent scRNA-seq analysis. For the chronic and sub-chronic smoke models, a single pool of cells was prepared per experimental group by pooling equal amounts of cells per animal of the respective group. Similarly, a single pool of cells was prepared from the negative control animals of the acute smoke model due to low cell counts whereas three biological replicates composed of three to four animals were prepared for both the smoke-exposed group and the treatment group (**Table 1**). Cell pools were washed once with HBSS/BSA/EDTA buffer followed by an additional wash with HBSS/BSA buffer (0.04% BSA in HBSS). Finally, cells were resuspended in HBSS/BSA buffer, filtered through a 40 µm cell strainer and collected in 2 mL low-bind Eppendorf tubes. All centrifugation steps were performed for 5 min at 320 g and 4°C.

**Table 1:** Sample overview.

Smoke model	Group	Animals per group	Number of BALF pools	Animals per BALF pool	Number of lung pools	Animals per lung pool
**Acute**	Air Smoke Treatment	7 11 11	1 3 3	7 4, 4, 3 4, 4, 3	3 3 3	2, 2, 3 4, 4, 3 4, 4, 3
**Sub-chronic**	Air Smoke Treatment	4 8 8	1 1 1	4 8 8	N/A	N/A
**Chronic**	Air Smoke	4 7	1 1	4 7	N/A	N/A

Overview of the experimental groups per smoke model and the corresponding cell pools prepared for single-cell RNA-sequencing. Animals were either exposed to room air (Air), cigarette smoke (Smoke) or cigarette smoke and treated with the IRAK4 inhibitor (Treatment). Cells from bronchoalveolar lavage fluid (BALF) were investigated for all model systems whereas lung tissue was only investigated for the acute smoke model. BALF and lung tissue pools of smoke-exposed and treated animals from the acute smoke model were prepared from the same animals, respectively.

### 2.5 Cell isolation from lung tissue

Lungs of the acute smoke model animals were removed after bronchoalveolar lavage and briefly cleaned in HBSS buffer. The diaphragm and trachea were removed and lungs were dissociated. Dissociation was performed using a gentleMACS™ Octo Dissociator (Miltenyi Biotec, Bergisch Gladbach, Germany) and the mouse Lung Dissociation Kit (Miltenyi Biotec, Bergisch Gladbach, Germany) as described by the manufacturer (standard program 37C_m_LDK_1). Lung tissue homogenates of the smoke-exposed and treated animals were then pooled according to the pooling strategy of the BALF samples. For the air-exposed group, three pools were prepared by pooling twice two animals and once three animals (**Table 1**). Pooled homogenates were filtered through a 70 µm cell strainer and washed twice in 10 mL of HBSS/BSA/EDTA buffer. Cell pellets were resuspended in 7.5 mL of buffer if two animals were pooled or 15 mL of buffer if more than two animals were pooled. Lung immune cells were then enriched by slowly adding 7.5 mL of resuspended cells on top of 6 mL Lymphocyte Separation Medium (Lonza, Basel, Switzerland). The mixture was then centrifuged for 20 min at 400 g and 4°C. The interphase containing immune cells was carefully transferred (interphases of both gradients if necessary) and washed twice in HBSS/BSA/EDTA buffer. Finally, the cells were washed once in HBSS/BSA buffer, resuspended, flushed through a 40 µm cell strainer and collected in 2 mL low-bind Eppendorf tubes. All centrifugation steps were performed for 5 min at 320 g and 4°C, unless specified otherwise.

### 2.6 Single-cell RNA-sequencing

The 10x Genomics Chromium™ system was used for scRNA-seq of the acute and sub-chronic smoke models and the Drop-seq platform was used for the chronic smoke model. In order to prepare scRNA-seq libraries via the Chromium™ system, 8,700 cells per sample were loaded onto the Chromium™ Controller (10x Genomics, Pleasanton, CA, USA) to capture 5,000 cells. Operation of the Chromium™ Controller and library preparation were performed as described by the manufacturer using the Chromium™ Single Cell A Chip Kit, Chromium™ Single Cell 3’ Library & Gel Bead Kits v2 and Chromium™ i7 Multiplex Kit (10x Genomics, Pleasanton, CA, USA). Quality of both the amplified cDNA and the final libraries was assessed by a Fragment Analyzer and the High Sensitivity NGS Fragment 1-6000 bp Assay (Agilent Technologies, Santa Clara, CA USA). Libraries were sequenced on a HiSeq 4000 device (Illumina, San Diego, CA) using a HiSeq 3000/4000 PE Cluster Kit and two 50-cycle SBS kits (Illumina, San Diego, CA), reaching an average mean depth of 61,000 (16% CV) and 50,000 (5% CV) mapped reads per cell per sample for the acute and sub-chronic models, respectively. The single index (8 bp) paired-end sequencing run comprised a 26 bp or 28 bp read 1 (cell barcode and UMI) and a 90 bp read 2 (transcript read).

Drop-seq was performed by means of a commercially available system (Dolomite Bio, Royston, UK) following the instructions of the manufacturer. For droplet generation, cell suspensions and barcoded beads (Barcoded Bead SeqB, ChemGenes Corp., Wilmington, MA, USA) were diluted to 300 cells/µL and 300 beads/µL, respectively. Further processing of the droplets and preparation of the sequencing libraries were performed as previously described (24). In total, 84,000 and 66,000 captured beads were used for cDNA amplification of the air control and smoke-exposed cell sample, respectively. A total of 670 pg cDNA was used to prepare the libraries via the Nextera XT DNA sample preparation kit (Illumina, San Diego, CA) and the custom primers and indices described by Macosko et al. (25). Libraries were sequenced at an average mean depth of 91,000 mapped reads per cell per sample (35% CV) on the HiSeq 4000 device as described above.

### 2.7 Data pre-processing

For both Drop-seq and 10X data, the transcript reads were trimmed to 90 bp. STARSolo v2.7.1a was used to align the reads back to the reference genome mm10, correct UMIs and cell barcodes, and to generate the raw count matrix as explained by the authors (https://github.com/alexdobin/STAR/blob/master/docs/STARsolo.md). Reads were annotated according to Ensembl v86. The count matrices were then used for down-stream analyses after removal of globin transcripts (*Hbb*- and *Hba*- transcripts). Quality control and down-stream analyses were performed using the Seurat R package (v3.0.0.9000) (26) unless stated otherwise.

### 2.8 Data filtering, downstream analysis and cell type annotation

Cells were kept for downstream analysis if mitochondrial transcripts represented < 10% of the total transcripts and between 300 and 5,000 genes were detected for the respective cell. The lower cut-off was set to a relatively low gene count to avoid removal of granulocytes that are characterized by lower gene counts compared to different cell types such as macrophages. In addition, genes detected in > 3 cells of the respective dataset were retained for further analysis. The filtered count matrices were used for subsequent downstream analysis of the acute, sub-chronic and chronic smoke model datasets.

#### 2.8.1 Acute smoke model

For the acute smoke model, we first analyzed the BALF and lung tissue samples from the smoke-exposed and air control animals. Transcript counts were normalized to 10,000 counts per cell and log-normalized using the “LogNormalize” function of the Seurat package. The highly variable genes of the merged dataset were then determined by Seurat’s “FindVariableFeatures” function using the “mean.var.plot” method (mean gene expression cut-offs: 0.0125 - 6, dispersion ≥ 0.5). Next, data were scaled using the “ScaleData” function with variances in UMI count being regressed out. Following principal component (PC) analysis, cell clusters were determined via Seurat’s default graph-based clustering strategy using the “FindNeighbors” and “FindClusters” functions. The first 48 PCs and a resolution of 1.5 were used to compute the cell clusters. Forty-three cell clusters were identified in the dataset and represented via UMAP that was also calculated on the first 48 PCs.

**Figure 2:**
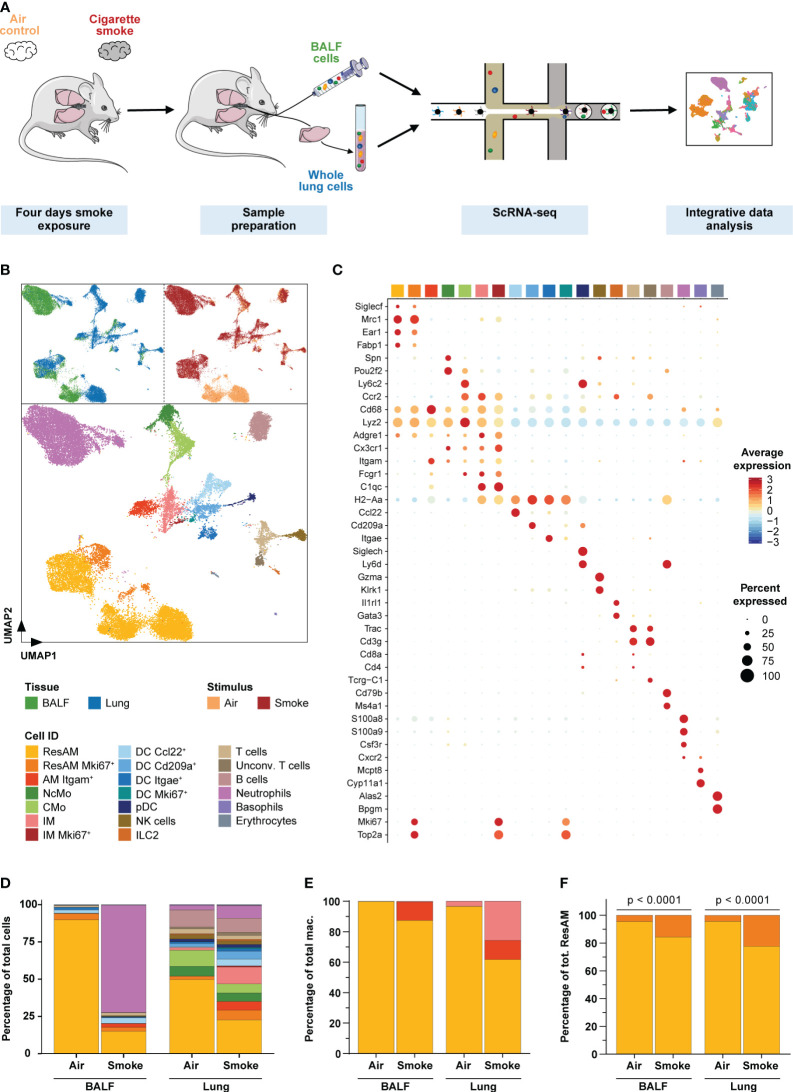
Single-cell characterization of pulmonary immune cells. **(A)** Experimental design. Mice were exposed to cigarette smoke for four days (n = 11) or air (n = 7; air control). Single-cell RNA-sequencing was performed on single-cell suspensions of pooled dissociated whole lung tissues and BALF for each experimental group (using equal cell counts per animal). ScRNA-seq data of both conditions and tissues were then jointly analyzed. **(B)** Single-cell representation of pulmonary immune cells using the first two components after dimensional reduction via Uniform Manifold Approximation and Projection (UMAP), colored by tissue (upper left panel), experimental condition (upper right panel) and annotated by immune cell types (bottom panel). **(C)** Scaled average expression of the canonical marker genes used to annotate immune cell clusters. Size of the dots represents the percentage of cells per cell type that express the respective gene. Identity of the cell types matches the colors used in the UMAP. **(D)** Relative frequency of the different immune cell populations in lung tissue and BALF of smoke-exposed and air control animals. **(E, F)** Relative frequency of the three main macrophage populations (irrespective of proliferation status) within the total macrophage population **(E)** and proliferating (Mki67^+^) as well as non-proliferating resident alveolar macrophages (ResAM) within the total ResAM population **(F)** in lung tissue and BALF of smoke-exposed and air control animals. Macrophage identity matches the colors used in the UMAP plot. Statistical significance was determined by Fisher’s test **(F)** on the absolute cell counts as determined by scRNA-seq (contingency tables are provided in **Supplementary Table S2**). AM, alveolar macrophages; BALF, bronchoalveolar lavage fluid; CMo, classical monocytes; DC, dendritic cells; ILC2, group 2 innate lymphoid cells; IM, interstitial macrophages; NcMo, non-classical monocytes; NK cells, natural killer cells; pDC, plasmacytoid DC.

Cell types were then annotated by a stepwise approach. Firstly, immune cell and non-immune cell clusters were identified based on CD45 expression as well as known immune cell and non-immune cell markers (**Figure 2C**, **Supplementary Figures S1A, D**). Two clusters co-expressed immune and non-immune cell markers and represented cell multiplets that were removed from further analysis. Secondly, immune and non-immune cell clusters were separately re-clustered (**Supplementary Figures S1B, C**). Cell clusters and UMAPs were calculated as described above using the first 36 PCs and a resolution of 1.5 for the immune cells and the first 35 PCs and a resolution of 1.4 for the non-immune cells. Cell types were then assigned to the cell clusters based on the expression of well-known cell type marker genes (**Figure 2C**, **Supplementary Figure S1D**). Clusters composed of spatially separated subclusters in UMAP representation were re-clustered to allow proper cell type annotation using the marker gene-based approach (**Supplementary Figure S1B, C**). Two low signal clusters of the immune cell dataset with a high ribosomal gene signature and/or low UMI count as well as clusters representing cell multiplets were excluded to ensure subsequent analysis on high-quality cells (**Supplementary Figure S1B**).

For focused analysis of neutrophils and the CMo, IM and MoAM fraction the respective cells were extracted from the immune cell dataset and separately analyzed following the procedure described above. Cluster analysis of the neutrophils and the CMo/macrophage fraction was performed on PC 1:15 and PC 1:14 and a resolution of 0.2 and 0.9, respectively. Further in-depth investigation of the identified IM cluster (PC 1:4, 0.15 resolution) and the lung tissue derived neutrophil cluster (PC 1:30, 0.3 resolution) revealed two biologically relevant subpopulations (N1, N2 and IM MHC-II^+^, IM Lyve1^+^). These subpopulations were accordingly annotated for subsequent analyses whereas a small contaminating cell multiplet cluster (1.2% of all cells) that co-expressed monocyte/macrophage and endothelial cell markers was excluded from the CMo/macrophage dataset.

Filtered and processed datasets of the treated animals were finally integrated into the analysis of air control and smoke-exposed animals (**Supplementary Figure S7A**). We made use of Seurat’s “FindTransferAnchors” and “TransferData” functions with default parameters to classify the cells from treated animals using the annotated dataset of the air and smoke controls as a reference. Low signal clusters characterized by a high ribosomal gene signature and/or low UMI count and cell multiplets were identified by cluster analysis and excluded from further analysis.

#### 2.8.2 Analysis and integration of sub-chronic and chronic smoke model data

The Seurat pipeline applied to the acute smoke model data was also used to process the data of the sub-chronic and chronic smoke models. The cells of both datasets were then annotated as described above using Seurat’s “FindTransferAnchors” and “TransferData” functions and the comprehensively annotated dataset of the acute smoke model as a reference. The refence-based approach was complemented by cluster analysis to identify low-quality clusters and cell multiplets in the annotated datasets. This approach revealed two low signal clusters characterized by a high ribosomal gene signature and/or low UMI count in the sub-chronic smoke model dataset that were excluded from further analysis (**Supplementary Figure S7B**). Finally, BALF scRNA-seq data from air control and smoke-exposed samples of the acute, sub-chronic and chronic smoke models were integrated into a single object for integrative analysis. Data integration was performed using the canonical correlation analysis approach implemented in the Seurat package via the “FindIntegrationAnchors” and “IntegrateData” functions. Default parameters and PC 1:31 were selected for data integration.

### 2.9 Differential expression analysis

Differential expression analysis to determine marker genes for a cell cluster compared to all remaining cells was calculated via the “FindAllMarkers” function while pairwise comparisons were performed using the “FindMarkers” function. Both functions utilized the MAST R package (v1.6.1) (27), which applies a generalized linear model approach that allows consideration of cellular detection rate. Only genes detected in ≥ 10% of the cells in at least one of the compared populations were considered for differential expression analysis. For the comparison of neutrophils from IRAK4 inhibitor treated and smoke-exposed animals a 5% cutoff was applied to account for treatment effects induced by shifts in rare subpopulations. Genes with absolute fold-changes (FC) ≥ 1.5 and an adj. *P* < 0.05 (Bonferroni correction) were considered significantly differentially expressed.

### 2.10 Trajectory analysis

Trajectory analysis of CMo, MoAM and IM was performed by Monocle (v2.8.0) (28) using the highly variable genes identified by Seurat. Single-cell trajectories were constructed by reducing data dimensionality via the “reduceDimension” function and “DDRTree” method and subsequent ordering of the cells along pseudotime using the “orderCells” function. The trajectory root was specified as the segment that comprised most CMo1 cells. Monocle’s “plot_genes_branched_pseudotime” function was used to visualize kinetic trends of selected genes along pseudotime per identified branch.

### 2.11 Gene set enrichment and network analysis

Gene set enrichment analysis (GSEA) was conducted by means of the gProfiler webtool (29) using the default settings. Interaction networks of the proteins encoded by selected gene sets were built through STRING (v11.0) (30) database using experimental, database, co-expression and text mining derived interaction sources and visualized by Cytoscape (v3.8.2) (31). Disconnected nodes were removed from the networks.

### 2.12 Cell-cell interaction

Potential cytokine and chemokine-mediated cell-cell interaction was inferred utilizing receptor-ligand pairs annotated in the murine “Cytokine-cytokine receptor interaction” KEGG pathway map (mmu04060) (32). To identify acceptor cell types of MoAM-derived chemokines we queried the expression of the corresponding receptors across cell populations and accepted them as potential acceptor cells if ≥ 10% of the cells expressed the respective receptor.

### 2.13 Protein quantification

TNF-α, IL-1β, IL-6 and CCL3 levels were determined in cell-free BALF by means of the V-PLEX Proinflammatory Panel 1 Mouse Kit (Meso Scale Diagnostics, LLC, Rockville, MD). CCL2 and CCL7 were quantified by DuoSet^®^ ELISA Development Kit (R&D Systems, Minneapolis, MN) and SimpleStep ELISA^®^ Kit (Abcam, Cambridge, UK), respectively. All measurements were performed according to the manufacturer’s instructions.

### 2.14 Histological analyses

Microscopy of Masson-Trichrome stained lung sections was performed using a Zeiss AxioScan Z1 whole slide scanner (Carl Zeiss, Jena, Germany) under bright field illumination and a 20x objective at 0.22 µm/px to analyze inflammation in lung sections with deep learning-based scores. Whole slide scans were exported as tiff with 50% down sampling (0.44 µm/px). Tiff images were then cut into non-overlapping tiles with 512 px x 512 px and fed into a convolutional neural network (CNN) trained to classify the degree of inflammation as 0 (no inflammation), 1 (6-10 inflammatory cells on tile), 2 (11-20 inflammatory cells on tile), or 3 (> 20 inflammatory cells on tile) as described previously (33). The output of the CNN were probabilities *p_0_, p_1_, p_2,_ p_3_* for classes 0, 1, 2, and 3, from which the inflammation *I* score was computed as weighted sum, i.e.


$$I=p_{0}0+\;p_{1}1+\;p_{2}2+\;p_{3}3$$

For the whole lung section, the average of the inflammation scores of all tiles was used. Mean linear intercepts were determined via a proprietary image evaluation tool on the basis of Hsia et al. (34).

### 2.15 Lung function measurements

Mice were anesthetized by i.p. injection of pentobarbital sodium and xylazine (60 mg/kg and 2.5 mg/kg, respectively) and a tracheal cannula was inserted after reaching a state of deep anesthesia. Lung function measurements were then performed by means of the flexiVent FX system (SCIREQ Inc., Montreal, Canada) that was equipped with the FEV extension and operated using the flexiWare software v7.6. Mechanical ventilation of the animals was performed at a respiratory rate of 150 breaths/min, a tidal volume of 6.5 ml/kg, and a positive end-expiratory pressure of 3 cmH_2_O. Spontaneous respiration efforts throughout the measurement were repressed by i.v. injection of pancuronium bromide (0.8 mg/kg). Measurements were conducted three times per animal.

### 2.16 Analysis of human data

We used the processed and annotated scRNA-seq dataset from our recent COPD study (35) for translational analyses. Similarity of murine MoAM to human BALF cell populations was assessed by GSEA on the human MoAM marker gene orthologs using the AUCell R package (v1.4.1) (36). For GSEA, genes of the human cells were ranked by expression and the top 3% thereof were used to compute area under the curve scores per cell. The scores were then normalized to a maximum value of 1 and visualized by violin plots.

### 2.17 Statistical analysis

Statistical analysis was performed by GraphPad Prism (v8.3.0; GraphPad Software, San Diego, CA, USA) and R (v3.5). Details are provided in the figure legends.

### 2.18 Data visualization

Plots were generated using the Seurat, ggplot2 (v3.0.0) and pheatmap (v1.0.10) R packages or GraphPad Prism. Experimental design figures were generated using images provided by Servier Medical Art under the Creative Commons Attribution 3.0 Unported License (https://creativecommons.org/licenses/by/3.0/).

## 3 Results

### 3.1 Acute smoke exposure triggers neutrophilic inflammation and occurrence of a unique alveolar macrophage subset in the lung

To examine the effect of acute smoke exposure (4 days) on the pulmonary immune cell types and their molecular mechanisms, we performed scRNA-seq on cells collected by bronchoalveolar lavage from the alveolar space and from lung tissue of the same CS-exposed (n = 11) and air control (n = 7) animals (**Figure 2A**). Integrative analysis on the computationally merged datasets was then performed to allow comprehensive investigation of the immune cell population across both compartments (**Figure 2A, B**). After removal of low-quality and non-immune cells, 30,014 cells (**Supplementary Table S1**) were left for in-depth investigation. These cells were annotated based on computationally determined clusters using well-known marker genes (**Figures 2B, C**, **Supplementary Figures S1A–D**). We identified all major immune cell populations of the murine lung, including classical (CMo) and non-classical monocytes, dendritic cells (DC Ccl22^+^, DC Cd209a^+^, DC Itgae^+^, and proliferating DC (DC Mki67^+^)), plasmacytoid dendritic cells, NK cells, group 2 innate lymphoid cells, T cells, unconventional T cells, B cells, neutrophils, basophils and few erythrocytes. Additionally, we identified proliferating (*Mki67* ^+^) and non-proliferating AM and interstitial (IM) macrophages (**Figures 2C, D**).

Smoke exposure dramatically altered the immune cell population in both, the airspace and lung tissue although the effect differed considerably across the two compartments (**Figure 2D**). These smoke effects were consistently observed across all biological replicates that were therefore merged for subsequent analyses (**Supplementary Figure S1E**). Overall, the strongest changes in relative cell abundance were observed for macrophages and neutrophils. Smoking induced a strong recruitment of neutrophils into the airspace and represented 72% of the cells in bronchoalveolar lavage fluid (BALF) of smoke-exposed animals whereas they were entirely absent in air controls (**Figure 2D**). This was validated by fluorescence flow cytometry (**Supplementary Figure S1F**), which confirmed acute neutrophilic inflammation in the airspace of smoke-exposed animals. Comparatively in lung tissue, we observed only a moderate 2.8-fold relative increase of neutrophils in smoke-exposed animals. In contrast, a 6.8-fold increase of IM was detected in lung tissue of these animals where they accounted for 12% of the immune cells while they were as expected not detected in BALF (**Figures 2D, E**). The lack of IM in BALF confirmed that the BALF macrophage population was derived from the alveolar space/lung lumen and not contaminated by macrophages from the lung tissue and thus represented AM. The BALF macrophages comprised ResAM and an *Itgam^+^* (CD11b) AM (AM Itgam^+^) subset that expressed canonical macrophage markers (*Cd68*, *Lyz2*, *Adgre1*, *Fcgr1*) (19, 37, 38) but lacked known ResAM markers (*Siglec1* ^+^, *Mrc1* ^+^, *Ear1* ^+^, *Fabp1* ^+^, *Itgam* ^-^) (19, 37) (**Figures 2C–E**). Itgam^+^ AM were only present in smoke-exposed animals where they represented approximately 12% of the BALF macrophages (**Figure 2E**). These data suggest that Itgam^+^ AM and the increased proliferative capacity of ResAM, reflected by a significant increase (> 3-fold) of proliferating ResAM, contributed to the 2.4-fold increase of macrophages in BALF from smoke-exposed animals (**Figure 2F**, **Supplementary Figure S1F**).

Taken together, our data demonstrate that acute smoke-induced lung inflammation is characterized by a neutrophilic pattern and increased macrophage heterogeneity including a unique subset of Itgam^+^ AM.

### 3.2 Recruited neutrophils are functionally diverse and important regulators of acute smoke-induced lung inflammation

**Figure 3:**
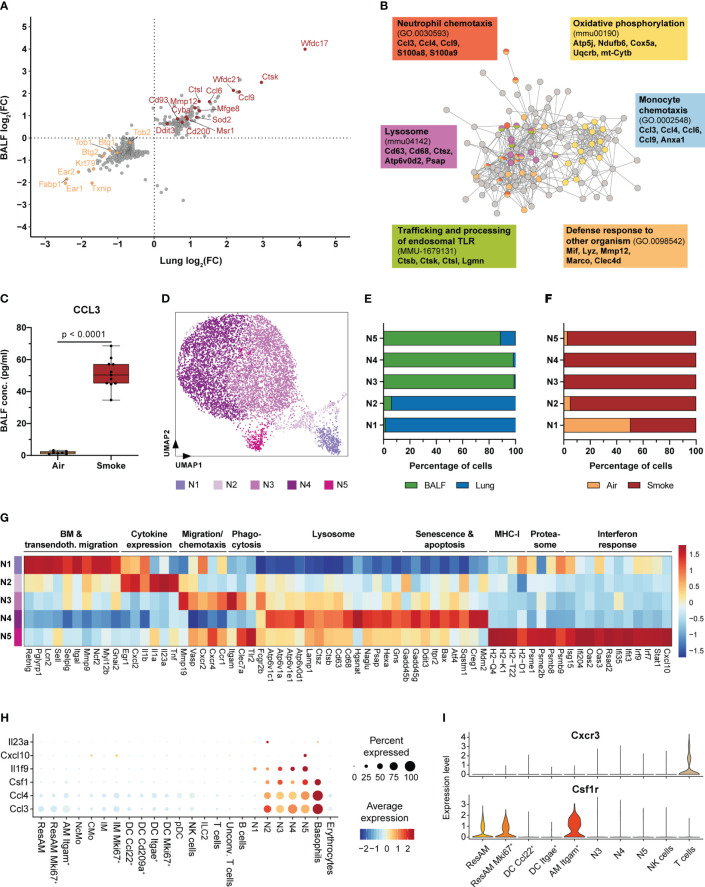
Neutrophils are key regulators of acute smoke-induced inflammation. **(A)** Comparison of smoke-induced alterations in gene expression between resident alveolar macrophages from BALF and lung tissue. Genes significantly up- and down-regulated by smoke (abs. fold change (FC) ≥ 1.5, adj. *P* < 0.05) in either lung, BALF or both tissues are displayed. Up- and down-regulated genes of interest are highlighted in red and orange, respectively. **(B)** Interaction network of the corresponding proteins encoded by the up-regulated genes shown in **(A)**. Nodes involved in selected significantly enriched (adj. *P* < 0.05) molecular pathways and processes are color-coded accordingly. Selected associated genes are listed per pathway or process. **(C)** BALF CCL3 levels in air controls (n = 7) and smoke-exposed mice (n = 11). Statistical significance was determined by Welch’s t-test. **(D)** UMAP representation of the five neutrophil subpopulations (N1-N5) identified by an independent cluster analysis of all neutrophils. **(E)** Fraction of cells derived from BALF or lung tissue within each neutrophil subpopulation. **(F)** Fraction of cells derived from smoke-exposed or control animals within each neutrophil subpopulation. **(G)** Molecular processes and average scaled expression levels of associated genes that are enriched across the different neutrophil subpopulations. **(H)** Dot plot representing the expression of pro-inflammatory markers that were predominantly expressed in neutrophils across all immune cell populations. Average scaled gene expression levels and the relative fraction of expressing cells are shown. **(I)** *Cxcr3* and *Csf1r* expression in BALF cell populations (cell populations that represented at least 0.5% of the BALF cell fraction). Panels **G-I** are based on smoke-exposed cells only.

Next, we investigated the transcriptomic profiles of neutrophils and AM to better understand their role in acute smoke-induced lung inflammation. In contrast to neutrophils and Itgam^+^ AM, ResAM were abundantly present in normal and smoke-exposed animals, which enabled direct comparison of their transcriptional profiles across both conditions (**Figures 2D**, **3A**). We identified 465 differentially expressed genes (abs. FC ≥ 1.5, adj. *P* < 0.05) in ResAM isolated from BALF and/or whole lung after smoke exposure that showed a highly similar response across both compartments (**Figure 3A**). ResAM of smoke-exposed animals were characterized by a decreased expression of anti-proliferative genes (*Btg1*, *Btg2*, *Tob1*, *Tob2*) (39–41), which is in line with their increased proliferative capacity (**Figures 3A**, **2F**). Smoke-exposed ResAM were also characterized by disturbed cellular homeostasis, illustrated by a decreased expression of genes related to homeostatic ResAM function (*Fabp1, Ear1, Ear2, Krt79*) (19), an up-regulation of oxidative phosphorylation associated genes (*Atp5j, Ndufb6, Ndufb2, Cox5a, Uqcrb, mt-Cytb*) and a de-regulation of genes involved in cellular and oxidative stress (*Ddit3*, *Txnip*, *Sod2*, *Cyba*) (**Figures 3A, B**). In addition, we detected an up-regulation of apoptotic cell clearance (*Msr1*, *Cd93*, *Mfge8*) (42–45) and lysosomal (*Cd63*, *Cd68*, *Atp6v0d2*, *Psap*) markers including cathepsins (*Ctsk*, *Ctsl*, *Ctsz*, *Ctsb*) that are also involved in endosomal TLR signaling (**Figures 3A, B**). Smoke-induced activation of defense related mechanisms was further supported by substantial induction of TLR-signaling associated *Wfdc21* and *Wfdc17* (46, 47) and genes that are linked to antimicrobial immunity (*Marco*, *Clec4d*, *Mif*, *Lyz2*, *Mmp12*) (**Figures 3A, B**). In agreement with activated host defense, we detected an increased expression of chemokines involved in neutrophil and monocyte/macrophage recruitment including *Ccl9*, *Ccl6*, *Ccl4* and *Ccl3* (**Figures 3A, B**). Increased CCL3 levels were also confirmed on the protein level in BALF of smoke-exposed animals, indicating that ResAM contributed to the recruitment of *Ccr1* ^+^ (encoding the CCL3 receptor CCR1) neutrophils into the alveolar space (**Figures 3C, G, H**).

Molecular characterization of neutrophils by cluster analysis identified five subclusters (N1-N5) that were characterized by a disproportional enrichment of neutrophils from the different experimental groups and lung compartments (**Figures 3D–F**). N1 and N2 neutrophils derived predominantly from whole lung, while clusters N3-N5 comprised mostly BALF neutrophils (**Figure 3E**). If separated by experimental group, air control neutrophils were mainly detected in the N1 cluster whereas neutrophils from smoke-exposed animals were found in all clusters (**Figure 3F**). Gene expression and GSEA identified distinct gene expression profiles among these different neutrophil subsets that were linked to a variety of molecular processes (**Supplementary Figure S2A**). Cluster N1 was characterized by a strong expression of genes elevated in bone marrow neutrophils (*Retnlg, Pglyrp1, Lcn2*) and associated with transendothelial migration (*Sell, Selplg, Itgal, Mmp9, Myl12b*), indicating that this cluster represented circulating neutrophils (48) (**Figure 3G**).

The N2 subset was in contrast characterized by the expression of pro-inflammatory chemokines and cytokines, such as *Cxcl2*, *Il1a*, *Il1b* and *Tnf*. In addition, the N2 subset was identified as the main source of *Il23a* (**Figure 3G**, **Supplementary Figure S2B**). This finding also matches the presence *Il23r* ^+^ unconventional T cells that expressed the proinflammatory down-stream mediator *Il17a* (49) (**Figure 3H**, **Supplementary Figure S2B**). Hence, the N2 neutrophil subset was identified as important regulator of the acute, smoke-induced pro-inflammatory environment in the lung. BALF neutrophil clusters N3 and N5 showed an increased expression of migration and chemotaxis associated markers (*Mmp19*, *Vasp*, *Cxcr4*) including the *Cxcl2* and *Ccl3* receptors *Cxcr2* and *Ccr1* (**Figure 3G**). In addition, these clusters were characterized by an increased expression of phagocytosis and pathogen clearance associated markers (*Clec7a*, *Itgam* and *Tlr2*) whereas the N4 subset was characterized by an increased expression of lysosomal markers (*Atp6v1c1*, *Cd68*, *Hgsnat*, *Psap*, *Hexa*) (**Figure 3G**). Furthermore, N4 neutrophils showed an increased expression of cellular stress, senescence and apoptosis markers (*Gadd45b*, *Gadd45g*, *Ddit3*, *Bax*, *Creg1*, *Mdm2*), suggesting that they represent aged neutrophils whereas N3 and N5 neutrophils seem to be more lately recruited (**Figure 3G**). Unlike the remaining neutrophil subsets, N5 neutrophils showed a prominent expression of MHC-I (*H2-Q4*, *H2-K1, H2-T22*, *H2-D1*), proteasomal (*Psme1*, *Psme2b*, *Psmb8, Psmb9*) and interferon response signature markers (*Ifi204*, *Oas2*, *Rsad2*, *Irf7*, *Irf9*) (**Figure 3G**). Despite the N5 subset accounted for only 3.5% of the BALF neutrophils in smoke-exposed animals, it plays a role in linking innate with adaptive immunity within the alveolar space. This is exemplified by characteristic *Cxcl10* expression of N5 neutrophils and the expression of its receptor *Cxcr3* in a subset of T cells (**Figures 3H, I**, **Supplementary Figure S2C**).

In summary, our data reveal that acute smoke exposure triggers host defense mechanisms and the expression of a few pro-inflammatory chemokines by ResAM, thereby contributing to the initiation of pulmonary inflammation. Neutrophils were characterized by functional heterogeneity, including an interferon response driven subset, and identified as important early regulators and amplifiers of pulmonary inflammation. This is illustrated by their strong expression of pro-inflammatory mediators including chemokines (e.g. *Ccl3*, *Ccl4*) and members of the IL-1 family, such as *Il1f9* (encoding for IL-36γ) that was specifically expressed by neutrophils and recently identified as an amplifier of pulmonary inflammation (50) (**Figure 3H**). Neutrophils also strongly expressed *Csf1* (encoding for macrophage colony-stimulating factor 1 (M-CSF)) that was recently shown to be required for the maintenance of MoAM in pulmonary fibrosis models (19). Interestingly, Itgam^+^ AM showed a significant, 1.7-fold increased *Csf1r* expression compared to ResAM, indicating a stronger dependence of this AM subset on M-CSF (**Figure 3I**). This observation and the absence of Itgam^+^ AM under homeostasis suggested that they represent MoAM.

### 3.3 Classical monocytes replenish the AM pool in smoke-induced lung inflammation

**Figure 4:**
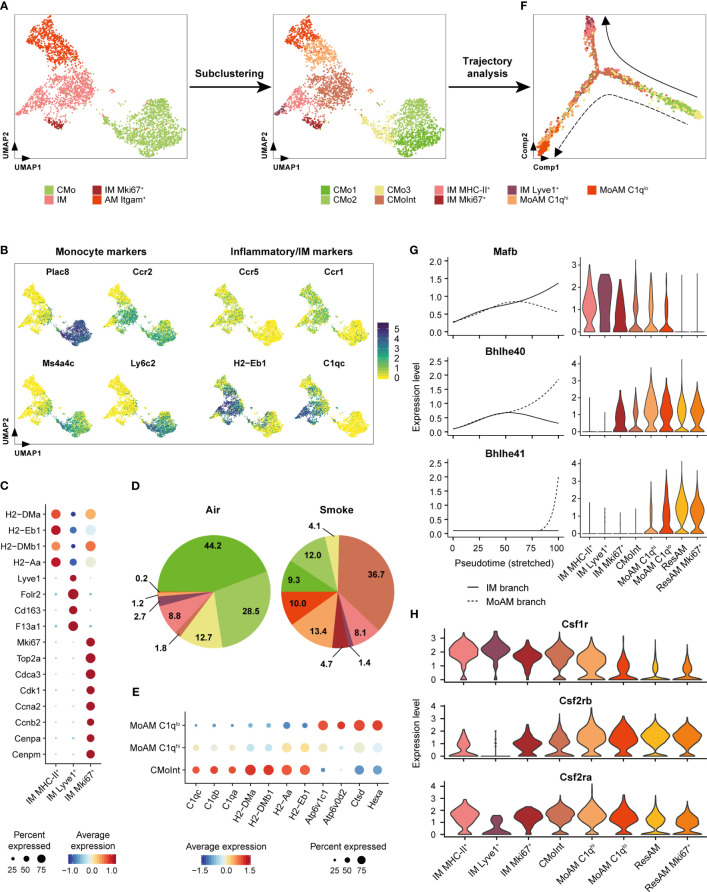
Itgam^+^ alveolar macrophages originate from inflammatory classical monocytes. **(A)** Subcluster analysis identifies cell subpopulations within the classical monocyte, interstitial macrophage and Itgam^+^ alveolar macrophage clusters including a monocyte/macrophage intermediate (CMoInt) population and monocyte-derived alveolar macrophage subsets (MoAM). **(B)** FeaturePlots display normalized expression levels of CMo marker genes and inflammatory/IM markers. Normalized expression levels are shown per single cell. **(C)** Dot plot displays average scaled expression levels of cluster-defining marker genes and fraction of expressing cells across the different IM subpopulations. **(D)** Relative frequency of the CMo, IM and MoAM subpopulations in whole lung of air control and smoke-exposed animals, respectively. Cell clusters are color-coded according to **(A)**. **(E)** Expression of MoAM subset defining markers across both MoAM subpopulations and CMoInt. Average scaled expression levels are color-coded and the dot size indicates the fraction of expressing cells per population. **(F)** Developmental trajectory analysis of the CMo, MoAM and IM subpopulations. Cell clusters are color-coded according to **(A)**. Dashed and solid arrows indicate the direction of differentiation into a MoAM (dashed line) and IM (solid line) lineage, respectively. **(G)** Expression of branch-dependent transcription factors is shown in the left panel as a function of pseudotime for the IM (solid line) and MoAM (dashed line) branch. Violin plots on the right panel show the expression levels of the same transcription factors across the different macrophage subpopulations as determined by cluster analysis and ResAM. **(H)** Violin plots show expression levels of selected growth factor receptors across the different macrophage subpopulations. Plots are based on cells from smoke-exposed animals only.

Dimensionality reduction analysis on the pulmonary immune cell population suggested a potential link between the CMo and IM clusters, which is supported by previous studies demonstrating CMo differentiation into IM as well as MoAM (51, 52) (**Figure 2B**). To further investigate the relationship between CMo and these macrophages, we performed cluster and trajectory analysis on this cell subset. Re-clustering subdivided the IM cluster into four transcriptionally distinct populations including *Lyve1* ^+^ (IM Lyve1^+^) and MHC-II^+^ (IM MHC-II^+^) IM as previously identified in the lung (**Figures 4A, C**) (19, 51, 53). In addition to these known IM subsets, we identified a cluster characterized by the expression of the proliferation marker *Mki67* and additional cell cycle regulating markers (*Cdk1, Ccnb2, Cenpa, Cdca3)* (**Figures 4A, C**). This IM Mki67^+^ subset was only detected in smoke-exposed animals, which supports the presence of IM with proliferative capacity in the smoke-induced inflammatory environment (**Figure 4D**). Besides the IM clusters, we identified an intermediate cell cluster called CMoInt that expressed several CMo and monocyte markers, such as *Plac8*, *Ccr2*, *Ms4a4c* and *Ly6c2*, illustrating that these cells originated from CMo (**Figures 4A, B**, **2C**). However, unlike CMo, CMoInt expressed inflammatory chemokine receptors, such as *Ccr1* and *Ccr5* (54–56) and increased massively in the lung of smoke-exposed animals. This is consistent with the concept of CMo recruitment during lung inflammation (**Figures 4B, D**). Finally, the common expression of MHC-II and C1q complement components by CMoInt, IM and Itgam^+^ AM corroborated that CMoInt represent an activated CMo-derived intermediate state (**Figures 4B, E**).

To examine developmental relationships of the re-clustered cell populations, we performed single-cell trajectory analysis on their gene expression profiles (**Figure 4F**). Superimposing the cluster IDs onto the pseudotime trajectory further substantiated that the CMoInt cluster comprised transitioning cells in between CMo and IM, which is concordant with a previous fate mapping study demonstrating that CMo replace Lyve1^+^ and MHC-II^+^ IM (**Figure 4F**) (51). However, a fraction of this intermediate cluster branches out towards Itgam^+^ AM, forming a second trajectory. This finally substantiated a CMo origin of the Itgam^+^ AM subset (**Figure 4F**). Consequently, we refer to this AM subset as MoAM hereafter. Re-clustering subdivided the MoAM population into two clusters referred to as C1q^lo^ MoAM and C1q^hi^ MoAM that were both detected in BALF (**Figure 4A**, **Supplementary Figure S3A**). In support of a potentially less mature state, C1q^hi^ MoAM were arranged along the MoAM trajectory branch and characterized by an increased expression of MHC-II and C1q complement components compared to C1q^lo^ MoAM. In contrast, the C1q^lo^ subset occupied the tip of the trajectory and displayed an increased expression of lysosomal markers (*Hexa, Ctsd, Atp6v0d2*), which indicates a more mature differentiation state (**Figures 4E, F**). In support of that, we detected a gradual decrease of the C1q regulating transcription factor *Mafb* (57) along the MoAM branch whereas the expression of *Bhlhe40/41*, which are central regulators of AM identity and repressors of *Mafb* (58), increased in the MoAM but not the IM branch (**Figure 4G**). The induction of transcriptional AM regulators was accompanied by a significant decrease (1.7-fold, adj. *P* = 4.1 x 10^-6^) of *Csf1r* expression in C1q^lo^ compared to C1q^hi^ MoAM whereas *Csf2rb* and *Csf2ra* (encoding for granulocyte macrophage colony-stimulating factor (GM-CSF) receptor chains) remained robustly expressed (**Figure 4H**). This indicates a loss of M-CSF dependence during MoAM maturation and finally their dependence on GM-CSF that is known to regulate AM function (59) and in line with the robust GM-CSF receptor expression by ResAM (**Figure 4H**). Nevertheless, characteristic ResAM markers were barely expressed by C1q^lo^ MoAM, which confirmed that this more mature MoAM subset remained molecularly distinct from ResAM (**Supplementary Figure S3B**).

Collectively, these data indicate CMo recruitment in smoke-induced lung inflammation and their differentiation into IM and MoAM under niche-specific transcriptional regulation.

### 3.4 MoAM are key drivers of smoke-induced lung inflammation and characterized by a tissue remodeling associated gene signature

**Figure 5:**
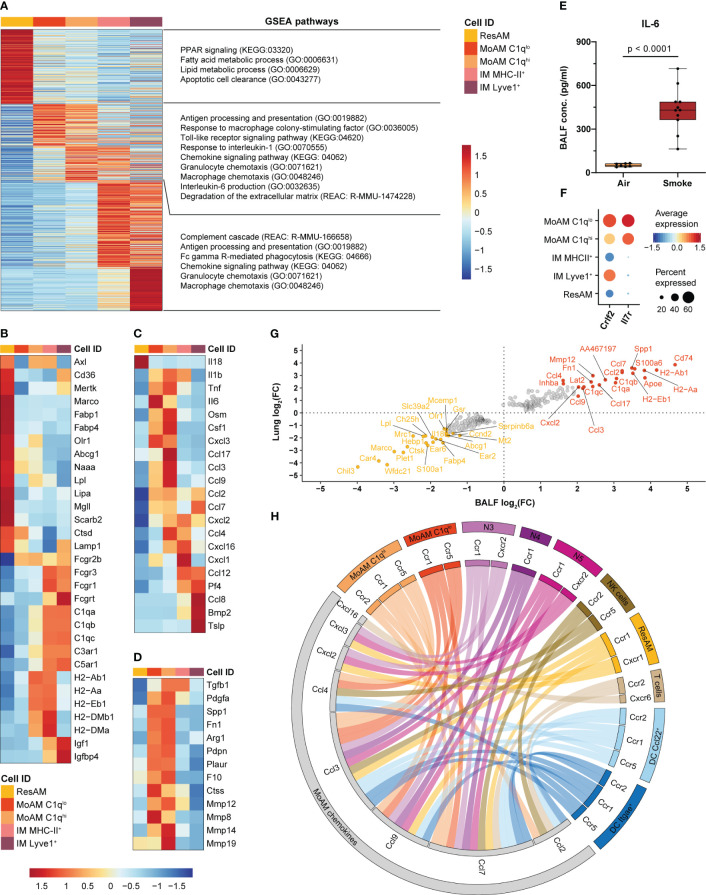
MoAM are key drivers of pulmonary inflammation and linked to tissue remodeling. **(A)** Heatmap representing the relative average expression of genes that are significantly differentially expressed (FC ≥ 1.5, adj. *P* < 0.05) across ResAM, MoAM and IM (sub)populations from smoke-exposed animals. Selected pathways that are significantly (adj. *P* < 0.05) associated with the respective macrophage (sub)population as determined by gene set enrichment analysis (GSEA) are listed on the right. **(B–D)** Expression of markers associated with the molecular pathways listed in **(A)** across the different macrophage (sub)populations. Heatmaps display the relative expression of gene panels that are related to ResAM and IM associated processes **(B)**, inflammatory markers **(C)** and tissue remodeling **(D)**. **(E)** IL-6 levels in BALF of air control (n = 7) and smoke-exposed (n = 11) animals. Statistical significance was determined by Welch’s t-test. **(F)** Dot plot illustrating the relative expression of the thymic stromal lymphopoietin receptor subunits *Crlf2* and *Il7r* across macrophage (sub)populations. **(G)** Scatter plot showing fold changes of the genes that were significantly differentially expressed between MoAM and ResAM in whole lung and BALF samples. The top 20 up- and down-regulated genes in BALF are annotated. **(H)** Circos plot depicting potential chemokine-mediated cell-cell interactions of MoAMs in the alveolar space. Chemokines that were significantly increased in MoAM compared to ResAM are connected with their receptors found across the different BALF cell populations (cell populations representing ≥ 0.5% of the BALF cell fraction and receptors expressed in ≥ 10% of the respective cell population are shown).

We next analyzed the gene expression profiles of MoAM specifically, to distinguish their role from that of the remaining macrophage populations. Differential gene expression analysis identified a transcriptional pattern unique to MoAM, which separated them from ResAM and IM (**Figure 5A**). GSEA was then performed on the differentially expressed gene sets to delineate functional differences of these macrophage populations during smoke-induced inflammation.

In comparison to AM, IM were characterized by an increased expression of Fc gamma receptors, complement and insulin-like growth factor signaling associated markers (*Fcgr3, Fcgr1, C5ar1, C1qb, Igf1, Igfbp4*). ResAM showed overall an increased expression of genes associated with canonical AM processes, such as fatty acid and lipid metabolism, PPAR signaling, phagocytosis, apoptotic cell clearance and lysosomal markers (*Olr1, Fabp4, Fabp1, Abcg1, Lpl, Lipa, Mgll, Naaa, Marco, Cd36, Axl, Mertk, Lamp1, Scarb2, Ctsd*) (**Figures 5A, B**). In contrast, MHC-II markers were increased in MHC-II^+^ IM and C1q^hi^ MoAM compared to ResAM and ranked among the genes that were most strongly increased in MoAM compared to ResAM, which indicates a role of MoAM in antigen presentation in the alveolar space (**Figures 5A, B, G**). MoAM were also identified as key regulators of the smoke-induced inflammatory response. This is illustrated by an association of MoAM marker genes with various inflammatory signaling pathways such as TLR signaling, “Response to interleukin-1” and “Interleukin-6 production”. In line with that, MoAM showed the strongest expression of IL-6 members (*Il6* and *Osm*), *Tnf* and *Il1b* across these different macrophage populations (**Figure 5C**). We confirmed the increased expression of IL-6, TNF-α and IL-1β also on the protein level in BALF of smoke-exposed animals, which corroborated a role of these central inflammatory cytokines in smoke-induced lung inflammation (**Figure 5E**, **Supplementary Figure S4A**). Interestingly, MoAM were also characterized by a significantly increased *Csf1* expression, indicating that autocrine M-CSF signaling may contribute to their early maintenance in addition to neutrophil derived M-CSF (**Figures 5C**, **3H, I**). The increased inflammatory profile of MoAM compared to ResAM was further substantiated by an increased expression of several pro-inflammatory chemokines (*Ccl2, Ccl7, Ccl3, Ccl4, Ccl9, Ccl17, Cxcl2, Cxcl3, Cxcl16*) (**Figures 5C, G**). Similarly, characteristic chemokine expression profiles were observed for IM subpopulations (**Figure 5C**, **Supplementary Figure S4B**). Several chemokines ranked among the most strongly up-regulated genes in MoAM compared to ResAM. These genes included *Ccl2* and *Ccl7* that were approximately 10-fold increased in MoAM (**Figure 5G**).

Comparison across all cell types found in murine lung confirmed MoAM and IM as major sources of *Ccl2* and *Ccl7* expression (**Supplementary Figure S4B**). Finally, increased CCL2 and CCL7 levels were confirmed at the protein level in BALF of smoke-exposed animals while they were barely detected in air controls (**Supplementary Figure S4A**). We further characterized the chemokine-based cellular crosstalk between MoAM and the remaining cell populations isolated from BALF by receptor-ligand interaction analysis on the chemokines that were more strongly expressed in MoAM than ResAM using curated chemokine receptor-ligand pairs (32). This analysis suggests that MoAM broadly interact with most cell populations found in BALF, thereby contributing to their own as well as neutrophil recruitment into the alveolar space via the *Ccl2/Ccl7-Ccr2* and *Cxcl2/Cxcl3-Cxcr2* axes, respectively (**Figures 5A, H**). MoAM characteristically expressed *Il7r*, which encodes for a component of the thymic stromal lymphopoietin (*Tslp*) receptor. *Tslp* was expressed by Lyve1^+^ IM as well as non-immune cells and is known to play a role in chronic inflammatory diseases and airway remodeling (60, 61) (**Figure 5F**, **Supplementary Figure S4B**). This example further illustrates the unique capacity of MoAM to engage in disease-relevant molecular circuits. A role of MoAM in tissue remodeling was further supported by their increased expression of tissue remodeling associated markers (*Spp1, Pdgfa, Arg1, Fn1, Tgfb1, Plaur, Pdpn*) including several genes encoding for metalloproteinases that are associated with extracellular matrix (ECM) degradation and emphysema such as *Mmp8, Mmp14, Mmp19*, *Mmp12* (**Figures 5A, D, G**). Thus, our data demonstrate a different functional role of recruited MoAM compared to ResAM.

Taken together, we identified MoAM and ResAM as two transcriptionally distinct AM populations. MoAM were characterized by a distinct tissue remodeling and pro-inflammatory gene signature, with an increased expression of several pro-inflammatory chemokines. These findings identify MoAM as key regulators of acute smoke-induced pulmonary inflammation in the alveolar space.

### 3.5 Chronic lung inflammation and emphysema are associated with a relative increase of MoAM

**Figure 6:**
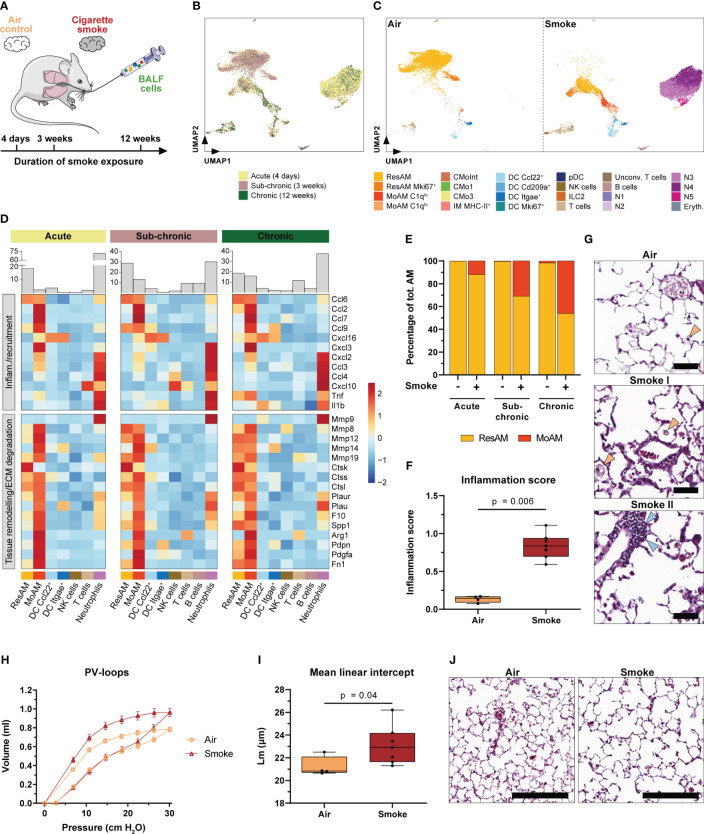
Accumulation of MoAM is linked to chronic pulmonary inflammation and emphysema. **(A)** Experimental design. BALF scRNA-seq datasets of mice exposed to sub-chronic (3 weeks) and chronic (12 weeks) smoke exposure were integrated into the acute (4 days) smoke model. **(B)** UMAP plot of the integrated BALF datasets. Cells are colored by duration of smoke exposure. **(C)** UMAP plot colored by cell (sub)population of air and smoke-exposed animals, left and right panel respectively. **(D)** Relative expression of genes encoding for selected pro-inflammatory chemokines and cytokines or genes associated with tissue remodeling/extracellular matrix (ECM) degradation (i.e. proteases and/or markers expressed by repair or remodeling associated macrophages). Relative gene expression is shown after acute, sub-chronic and chronic smoke exposure for the main cell populations (≥ 2% of the cells for at least one timepoint). Barplots located on top of the heatmap indicate the frequency of the different cell types for the respective model system. **(E)** Abundance of MoAM and ResAM within the total AM population identified in BALF of air control and smoke-exposed mice after acute, sub-chronic and chronic smoke exposure. **(F)** Inflammation scores of air control (n = 4) and smoke-exposed (n = 7) mice upon chronic smoke exposure. Inflammation scores were determined by a machine-learning based approach (see methods for details) using lung tissue sections (min = 0 (no inflammation), max = 3 (strong inflammation)). Statistical significance was determined by Mann-Whitney U test. **(G)** Representative Masson trichrome stained lung sections from air control and smoke-exposed mice after chronic smoke exposure. Orange arrows indicate alveolar macrophages increasing after smoke exposure whereas blue arrows indicate an immune cell infiltrate. Scale bar: 50 µm. **(H)** Pressure-volume (PV) loops of mice after chronic smoke exposure (n = 7) and the corresponding air controls (n = 4). Data points indicate mean +/- SD. **(I)** Mean linear intercept (Lm) of the same animals as shown in **(H)**. Statistical significance was determined by Mann-Whitney U test. **(J)** Masson trichrome stained lung sections after chronic smoke exposure and a corresponding air control indicating enlarged alveolar spaces upon smoke exposure. Scale bar: 200 µm.

In order to investigate the extent to which MoAM play a role during the further course of smoke-induced pulmonary inflammation and COPD-like pathology, we integrated BALF scRNA-seq datasets from sub-chronically (3 weeks) and chronically (12 weeks) smoke-exposed mice and the respective air controls into our analysis (**Figures 6A, B**). Overall, we identified the same cell populations in BALF of the sub-chronic (7,717 cells) and chronic (1,083 cells) models as observed during the acute state of smoke-induced inflammation, albeit at different ratios (**Figure 6B-D**, **Supplementary Table S1**). In agreement with the observations of the acute smoke model, MoAM and neutrophils were found after sub-chronic and chronic smoke exposure while they were virtually absent in air controls. This was consistent with a significant increase of macrophages and neutrophils in BALF of the smoke-exposed animals (**Figures 6C, E**, **Supplementary Figure S5A, B**). Although the relative frequency of neutrophils decreased in the later stages of smoke-induced lung inflammation, they remained a key source of pro-inflammatory mediators, as exemplified by the strong expression of various chemokines (*Cxcl2*, *Ccl3*, *Ccl4*, *Cxcl10*) as well as *Tnf* and *Il1b* (**Figure 6D**). Significantly increased levels of TNF-α and IL-1β were validated in BALF of chronically smoke-exposed animals (**Supplementary Figure S5D**).

To further explore the role of MoAM in sub-chronic and chronic smoke-induced inflammation, we compared their gene expression profile to those of the remaining cell types, particularly ResAM. As observed after acute smoke exposure, MoAM from sub-chronically and chronically smoke-exposed mice were characterized by a strong expression of pro-inflammatory chemokines (**Figure 6D**). Importantly, MoAM showed a significantly increased expression of neutrophil, T-cell and MoAM recruitment associated chemokines (*Ccl2*, *Ccl7*, *Ccl9*, *Cxcl16*, *Cxcl2*, *Cxcl3*, and *Ccl3*) compared to ResAM in at least two of the model systems (**Figure 6D**). The relative MoAM frequency in BALF increased from 2.4% after acute smoke-exposure to 12.9% and 16.1% after sub-chronic and chronic smoke exposure, respectively (**Figure 6D**). The increase of MoAM after extended smoke exposure was also related to an altered ResAM/MoAM ratio. While MoAM represented 12.0% of the total AM population after acute smoke exposure, their relative proportion increased substantially to 30.9% and 46.1% after sub-chronic and chronic smoke exposure, respectively (**Figure 6E**). The relative increase of MoAM within the AM pool was also associated with a relative increase of the more mature C1q^lo^ MoAM subpopulation (**Supplementary Figure S5C**). These data demonstrate that continuous, smoke-induced recruitment and accumulation of MoAM substantially alters the AM pool towards an increased inflammatory and tissue remodeling associated AM profile in the chronic state. This was accompanied by a phenotypically heterogeneous macrophage population in the alveolar space including foamy macrophages (**Figure 6G**). Collectively, these longitudinal observations indicate that MoAM also remain key regulators of the chronic smoke-induced inflammatory response and drive the development and maintenance of chronic pulmonary inflammation. The latter was phenotypically confirmed by histological assessment of chronically smoke-exposed mice, which showed significantly increased inflammation scores compared to air controls and perivascular immune cell infiltrates (**Figures 6F, G**).

Similar to MoAM isolated after acute smoke exposure, MoAM derived from the chronically smoke-exposed mice were characterized by a tissue remodeling associated gene expression profile, with several markers linked to the development of emphysema or to the cleavage of ECM components such as *Spp1*, *Mmp12*, *Mmp14, Mmp8*, *Mmp19*, *Ctsk* and *Ctss* (21, 62–66) (**Figure 6D**). In agreement with that finding and the relative increase of MoAM in chronically smoke-exposed mice, these animals were characterized by impaired lung function as demonstrated by an upward shift of the pressure-volume loop that is typically seen in emphysematous mice (**Figure 6H**) (67). Finally, histological analysis phenotypically confirmed pulmonary emphysema in these animals indicated by enlarged airspaces and significantly increased mean linear intercept length in smoke-exposed animals compared to air controls (**Figures 6I, J**). These data strongly support the involvement of MoAM in the development of emphysema based on their expression profile. Nevertheless, most of the tissue remodeling associated markers showed a similar expression in ResAM after chronic smoke exposure while they showed a relatively lower expression in ResAM during the acute state of smoke-induced pulmonary inflammation (**Figure 6D**).

In summary, our data identify MoAM and neutrophils as key regulators of acute and chronic smoke-induced lung inflammation in the alveolar space. Chronic pulmonary inflammation leads to emphysema and a relative increase of MoAM that are characterized by a tissue remodeling and emphysema associated gene expression profile.

### 3.6 Human MoAM orthologs are increased in emphysema patients

**Figure 7:**
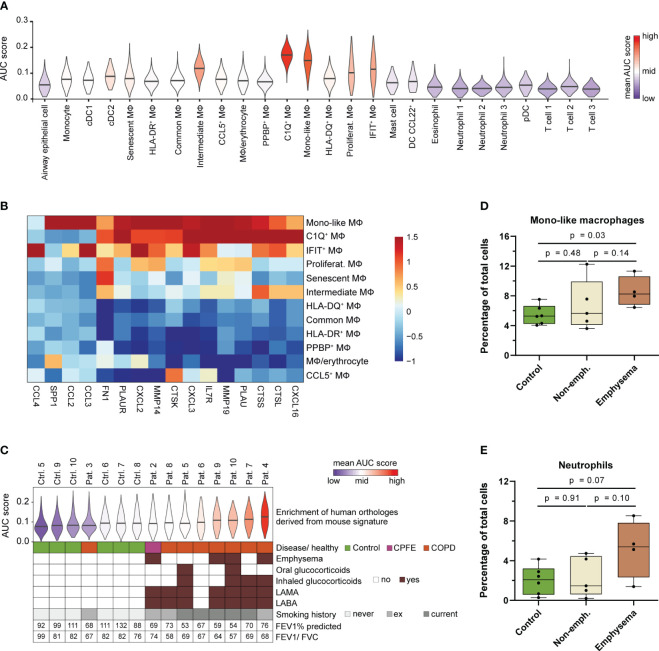
Human MoAM orthologs are increased in lung emphysema patients. **(A)** Enrichment of the murine MoAM signature genes (human orthologs of the chronic smoke model) across cell populations from human BALF. Human data are derived from a recent scRNA-seq study that includes chronic cough controls, emphysematous and non-emphysematous chronic obstructive pulmonary disease (COPD) as well as combined pulmonary fibrosis and emphysema (CPFE) patients (35). **(B)** Relative expression of murine MoAM associated pro-inflammatory and tissue remodeling related genes across the human BALF macrophage subpopulations. **(C)** Distribution of enrichment scores of the murine MoAM signature genes (human orthologs) within the macrophage population per human specimen. Additional clinically relevant information is provided per donor. **(D, E)** Abundance of mono-like macrophages and neutrophils in BALF of controls (n = 6), non-emphysematous (n = 5) and emphysematous (n = 4) patients. Statistical significance was determined by Kruskal-Wallis/Dunn’s multiple comparison test. AUC, area under the curve; FEV1/FVC, ratio of forced expiratory volume in one second to forced vital capacity; FEV1% predicted, FEV1 percentage of predicted normal; LABA, long acting beta antagonist; LAMA, long acting muscarinic antagonist; MΦ, macrophage.

To examine the observed link between MoAM, smoke-induced chronic inflammation and the development of pulmonary emphysema in humans, we performed enrichment analysis of the murine MoAM gene signature orthologs from the chronic smoke model across the BALF cell populations identified in our recently performed COPD study (**Figure 7A**) (35). As expected, an overall stronger enrichment of the MoAM gene signature was observed in human macrophage populations compared to the remaining cell types (**Figure 7A**). Among these macrophage subsets, C1Q^+^ and mono-like macrophages showed the strongest enrichment scores for the murine MoAM gene signature (**Figure 7A**). These human macrophage populations were also characterized by an increased expression of tissue remodeling and ECM degradation associated markers (*SPP1*, *MMP14*, *MMP19*, *FN1*, *PLAUR, IL7R, CTSL, CTSS, CTSK*) although they were not exclusively expressed by C1Q^+^ and mono-like macrophages (**Figure 7B**). Genes encoding for chemokines (*CXCL2*, *CXCL3*, *CXCL16*, *CCL3*, *CCL2*) involved in neutrophil and MoAM recruitment were also strongly expressed in these human macrophages, particularly human mono-like macrophages. Thus, MoAM orthologs with pro-inflammatory and tissue remodeling associated gene expression profiles are present in humans (**Figure 7B**).

We further investigated the macrophage fraction of the human control (chronic cough, never smokers) and patient subjects (emphysematous and non-emphysematous GOLD 2 COPD and combined pulmonary fibrosis and emphysema (CPFE) patients including current and ex-smokers) to assess MoAM relevance in COPD and emphysema (**Figure 7C**). The murine MoAM gene signature showed an overall increased enrichment for the macrophages isolated from COPD patients compared to controls. Ranking of the average enrichment score almost perfectly separated patients from control subjects (**Figure 7C**). Most emphysematous patients were among the top ranked subjects, suggesting an overall increased fraction of macrophages that express MoAM associated genes in this patient subset (**Figure 7C**). This was further substantiated by a significant increase of mono-like macrophages in emphysematous patients compared to controls whereas C1Q^+^ macrophages were not increased (**Figure 7D**, **Supplementary Figure S6**). In non-emphysematous patients, the relative frequency of mono-like macrophages was more variable and, overall, not increased compared to controls (**Figure 7D**). The increase of mono-like macrophages was accompanied by a trend towards an increased relative abundance of neutrophils in emphysematous patients compared to controls and non-emphysematous patients (**Figure 7E**).

Taken together, we identified an orthologous human MoAM population that is characterized by a tissue remodeling/ECM degradation and pro-inflammatory gene signature. This human MoAM population is enriched in humans with emphysema.

### 3.7 MoAM and neutrophil recruitment is diminished by anti-inflammatory IRAK4 inhibitor treatment

**Figure 8:**
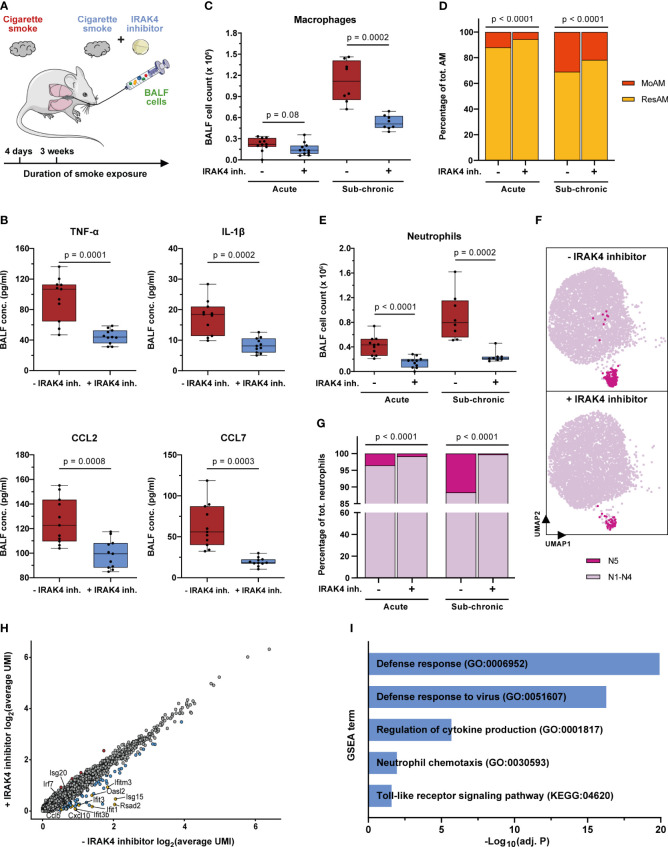
IRAK4 inhibition alleviates pulmonary inflammation and reduces the recruitment of MoAM and neutrophils. **(A)** Experimental design. The impact of IRAK4 inhibitor treatment on cells isolated from BALF was investigated after acute (4 days) and sub-chronic (3 weeks) smoke exposure. **(B)** TNF-α, IL-1β, CCL2 and CCL7 concentration in BALF of IRAK4 inhibitor treated and untreated mice after acute smoke exposure (n = 11 per group). **(C)** Macrophage counts in BALF of IRAK4 inhibitor treated and untreated mice after acute (n = 11 per group) and sub-chronic (n = 8 per group) smoke exposure as determined by fluorescence flow cytometry. **(D)** MoAM and ResAM frequency within the total BALF alveolar macrophage population upon IRAK4 inhibitor treatment after acute and sub-chronic smoke exposure as determined by scRNA-seq. **(E)** Neutrophil counts in BALF of treated and untreated mice after acute and sub-chronic smoke exposure as determined by fluorescence flow cytometry. **(F)** UMAP plot split by BALF neutrophils derived from acute smoke-exposed, untreated (-IRAK4 inhibitor) and smoke-exposed, treated (+IRAK4 inhibitor) mice. Cells of the antiviral neutrophil subpopulation N5 (**Figure 3G**) are highlighted and 5,000 cells are depicted per group. **(G)** Neutrophil frequency with and without IRAK4 inhibitor treatment after acute and sub-chronic smoke exposure as determined by scRNA-seq. **(H)** Correlation of the average gene expression levels from neutrophils of IRAK4 inhibitor treated and untreated mice after sub-chronic smoke exposure. Genes that are up- and down-regulated (adj. *P* < 0.05, abs. FC > 1.5) by IRAK4 inhibitor treatment are highlighted in red and blue, respectively; down-regulated genes of particular interest in this study have been highlighted in yellow. **(I)** Significantly enriched GSEA terms (adj. *P* < 0.05) associated with the significantly down-regulated genes in neutrophils of the treated compared to the untreated mice after sub-chronic smoke exposure. Statistical analysis: **(B)** Welch’s t-test, **(C, E)** Mann-Whitney U test **(D, G)** Fisher’s test on the absolute cell counts as determined by scRNA-seq (contingency tables are provided in **Supplementary Tables S3**, **S4**).

Our study demonstrates activation of pro-inflammatory and defense response mechanisms by smoke exposure, including TLR and IL-1 signaling (**Figures 3A, B, G**, **5A, C**, **6D**). This led to an aberrant, chronic pulmonary inflammation and finally emphysema. In mice and humans, emphysema was associated with a relative increase of MoAM that, together with neutrophils, were identified as key amplifiers of pulmonary inflammation making these cell types and inflammatory signaling pathways attractive therapeutic targets. Therefore, we examined in first proof-of-principle studies whether pharmacological inhibition of the IL-1R/TLR-signaling associated IRAK4 kinase alleviates pulmonary inflammation and affects the recruitment of neutrophils and MoAM upon acute and sub-chronic smoke exposure (**Figure 8A**).

IRAK4 inhibitor treatment throughout acute smoke exposure significantly decreased TNF-α and IL-1β (53% decrease, each) BALF levels which demonstrates the anti-inflammatory effect of the IRAK4 inhibitor (**Figure 8B**). Significantly decreased levels of the MoAM associated chemokines CCL2 and CCL7 (21% and 69%, respectively) and a strong trend towards decreased total BALF macrophage counts (30% decrease on average, p = 0.08) in treated animals further corroborates the anti-inflammatory effect of the IRAK4 inhibitor (**Figures 8B, C**). ScRNA-seq analysis revealed the same cell (sub)populations in BALF of treated animals and smoke controls (**Supplementary Figure S7A**). However, we observed a significant 53% decrease in relative MoAM frequency within the total AM population of treated animals compared to their smoke-exposed, untreated littermates (**Figure 8D**). These data indicate decreased MoAM recruitment in treated animals that was also confirmed when investigating the biological replicates analyzed after acute smoke exposure (**Supplementary Figure S7C**). Decreased MoAM recruitment was further substantiated by a significantly, 30% decreased relative MoAM frequency and on average 52% decline of total macrophage counts in treated animals after sub-chronic smoke exposure (**Figures 8C, D**, **Supplementary Figure S7C**).

Next, we evaluated whether IRAK4 inhibitor treatment also succeeded in reducing the recruitment of neutrophils. Treated animals showed on average a 64% and 73% decreased neutrophil count in BALF compared to non-treated controls after acute and sub-chronic smoke exposure, respectively (**Figure 8E**). ScRNA-seq based in-depth characterization of the neutrophil population further demonstrated a significant treatment effect on the rare interferon response driven neutrophil cluster (N5). IRAK4 inhibitor treatment reduced the relative abundance of this pro-inflammatory neutrophil subset by 77% representing only 0.8% of the BALF neutrophils in treated animals as compared to 3.5% in acute smoke-exposed controls. As observed for MoAM, the treatment effect on neutrophils was highly consistent across all biological replicates, which further corroborates the treatment effect on this rare cell population (**Figures 8F, G**, **Supplementary Figure S7D**). An even more pronounced decline of N5 neutrophils in treated animals was observed after sub-chronic smoke exposure, where they represented only 0.3% of the BALF neutrophils, which corresponds to a 97% decrease compared to smoke-exposed controls (**Figure 8G**). Congruently, differential gene expression analysis identified a significant down-regulation of N5 neutrophil associated genes within the total neutrophil population of treated compared to untreated animals. Several of these genes were linked to defense/antiviral response and TLR signaling (*Rsad2, Ifit1, Ifit3, Ifit3b, Ifitm3, Oasl2, Isg20, Irf7, Isg15, Cxcl10, Ccl5*) as well as neutrophil and T cell recruitment (*Ccl5, Isg15, Cxcl10*). This observation demonstrates that IRAK4 inhibition induced the depletion of N5 neutrophils and contributed to its anti-inflammatory effect on smoke-induced pulmonary inflammation (**Figures 8H, I**, **Supplementary Figure S7D**).

In summary, we demonstrate an anti-inflammatory effect of IRAK4 inhibitor treatment on smoke-induced pulmonary inflammation that is associated with a reduction of MoAM as well as neutrophil recruitment and their pro-inflammatory mediators. High-resolution scRNA-seq even resolved the depletion of the rare pro-inflammatory and interferon response-driven neutrophil subset in treated animals, which illustrates the power of such high-resolution transcriptomic analyses in pharmaceutical development.

## 4 Discussion

Globally, cigarette smoking is the single most avoidable cause of illness and death, as well as the leading risk factor for COPD in Western societies (4, 13). Despite chronic lung inflammation being a hallmark of the disease and key to COPD pathogenesis (9, 11), the cellular basis and molecular mechanisms underlying smoking-related lung inflammation and the progression from an acute to a chronic, pathologically relevant state remain poorly understood. Here, we used scRNA-seq to perform unbiased profiling of the immune cell landscape within the alveolar space/airspace of mice after acute, sub-chronic and chronic smoke exposure with a particular focus on neutrophils and AM in order to broaden our understanding of the cellular and molecular alterations associated with the different stages of smoke-induced lung inflammation and the development of COPD-like pathology, namely emphysema. Besides confirming the recruitment and increase of various immune cell populations in BALF as reported earlier (22, 68, 69), we discovered previously unknown cellular diversity, cellular dynamics and molecular responses throughout the course of smoke-induced lung inflammation. Importantly, we demonstrated considerable molecular and cellular alterations to the AM pool following smoke exposure characterized by the progressive increase of MoAM. These non-resident MoAM differ transcriptionally from resident AM, are key drivers of lung inflammation, and their increase is eventually associated with the development of emphysema. Cross-comparison with human scRNA-seq data (35) confirmed the increase of MoAM orthologs also in emphysematous COPD patients, which illustrates the power of such translational approaches to uncover so far unknown cellular alterations in the context of human diseases.

Direct involvement of MoAM in the development of emphysema and tissue remodeling is supported by their molecular profile including the expression of elastolytic/matrix degrading and emphysema-associated markers. Among these markers, *Mmp12* and osteopontin (*Spp1*) are essential for the development of smoke-induced emphysema in mice whereas MMP-12/SPP-1 levels in sputum were shown to correlate with the extent of emphysema in COPD patients (21, 66, 70, 71). Increased MMP-14 levels were also detected in emphysematous lungs of humans (63), which collectively corroborates a role of MoAM/mono-like macrophages in the development of pulmonary emphysema at the molecular level for both mice and human. Similarly, Shibata et al. recently demonstrated that the development of elastase-induced emphysema was dependent on the recruitment of Ly6C^hi^/CCR2^+^ monocytes using adoptive transfer studies and monocytes from wild type and CCR2-deficient mice (72). While this previous study confirmed the potential of CMo-derived macrophages to contribute to structural lung damage, the recruited CMo were shown to primarily differentiate into IM instead of AM (72). This contrasts with our study, which suggests that a considerable fraction of *Ccr2* ^+^ CMo differentiates via a common CMo/macrophage intermediate state into MoAM besides differentiating into IM. The dual fate of CMo as suggested by our study is supported by previous fate mapping studies demonstrating that Ly6C^hi^ CMo differentiate into IM and that AM recruited into the alveolar space following influenza infection originate from CCR2-dependent bone marrow-derived monocytes (17, 51). We hypothesize that cigarette smoke may provoke an increased differentiation of recruited CMo towards MoAM that contribute together with locally proliferating ResAM to the increase of macrophages in the alveolar space (22, 68) and finally to tissue damage. While the capacity for self-renewal is well established for ResAM it is thought to be impaired for IM (15, 73, 74). Our data challenge this concept together with a previous study (53) as we found evidence for a proliferating IM population that emerged in inflamed lungs. Whether this population represents resident or even monocyte-derived IM that become proliferative due to smoke-induced changes in their microenvironment needs to be clarified in future studies.

AM of monocytic origin have been recently shown to differ in their molecular response and function from ResAM during acute influenza infection and in models of pulmonary fibrosis (16, 17, 19). Here, we demonstrated that AM origin also dictates their response during smoke-induced pulmonary inflammation. Consistent with their function as first line defense against inhaled pathogens and particles (13, 75) our study supports a role of ResAM in the initiation of smoke-induced lung inflammation by contributing to neutrophil and monocyte/MoAM recruitment. However, the strong induction of negative regulators of inflammation in ResAM, such as *Wfdc17*/*21* (46, 47), also illustrates the presence of counter-regulatory mechanisms in these resident macrophages to avoid excessive inflammation, which is important to ensure gas exchange and avert exaggerated tissue damage in the lung (75). In contrast, we have shown that MoAM are characterized by a strong pro-inflammatory profile, which is in line with previous reports from bleomycin-induced lung injury and viral/bacterial infection models (16, 17, 76). However, these previous studies also demonstrated that MoAM recruitment is limited to the inflammatory window during an influenza infection and that recruited pro-inflammatory MoAM adopt a similar molecular profile as ResAM over time in the absence of subsequent inflammatory stimuli (16, 17). These data illustrate the importance of controlled MoAM recruitment and their molecular reprogramming following resolution of inflammation to re-establish pulmonary homeostasis. Our study indicated that smoking prevents controlled resolution of pulmonary inflammation, which triggers continuous recruitment and accumulation of pro-inflammatory MoAM as well as a progressive decrease of the ResAM/MoAM ratio in the alveolar space probably due to repetitive inhalative tissue injury (6). By applying unbiased scRNA-seq we were able to highlight the central role of MoAM in orchestrating and amplifying the smoke-induced inflammatory response in the alveolar space. This central role of MoAM is based on their increased expression of various pro-inflammatory mediators compared to the remaining immune cell populations in BALF, predominantly chemokines involved in the recruitment of inflammatory cells, such as MoAM, neutrophils and T-cells. While our longitudinal data suggest that both AM subsets eventually contribute to tissue damage, pro-inflammatory chemokine expression remained increased in MoAM even after 3 months of smoke exposure, corroborating the inherently impaired pro-inflammatory capabilities of ResAM compared to MoAM. Thus, our study suggests that chronic smoking establishes a self-amplifying inflammatory loop with MoAM as key inflammatory regulators leading to excessive chronic pulmonary inflammation and eventually emphysema.

Among the various pro-inflammatory mediators strongly expressed by MoAM several are linked to COPD including *CCL2* and *CCL7*, which have been shown to be increased in induced sputum of COPD patients (18, 77). Of note, both of them are expressed by small sputum macrophages in humans, which represents a phenotypically distinct macrophage population that was shown to be > 6-fold increased in sputum of COPD patients compared to healthy controls (77). Due to the characteristic expression of *Ccl2/Ccl7* by murine MoAM, we hypothesize that small sputum macrophages reflect their human orthologs and correspond to the human *CCL2* ^+^ mono-like macrophages that were increased in BALF of the emphysematous COPD patients. The central role of CCL2 and CCL7, and thus MoAM, in lung inflammation and pathology is corroborated by a strongly impaired recruitment of monocytes into the lung and protection from pulmonary emphysema upon inhibition of the CCR2/CCL2 axis (72, 78). Moreover, CCL2/CCL7 contribute to neutrophil recruitment into the airspace during LPS-induced inflammation (79); an observation that is conform with the chemokine-based cellular interaction network identified in BALF cells during smoke-induced pulmonary inflammation in this study.

We have shown that recruited neutrophils comprise functionally distinct subsets including a rare subset (N5) characterized by an interferon response signature, which has previously also been observed in lung cancer tissue of humans and mice (80). However, this neutrophil subset is decreased in lungs of tumor-bearing mice (80) whereas our study identified an increase in the alveolar space upon smoking. This illustrates the potential of high-resolution transcriptomic profiling to identify and differentiate the role of such molecular cell subsets across pathological conditions. Indeed, functional relevance of the N5 subset for smoke-induced lung pathology is supported by its strong expression of *Cxcl10*, which is increased in sputum of COPD patients and smokers and known to induce emphysema associated MMP-12 (81, 82). Moreover, cells positive for the CXCL10 receptor CXCR3 and CD8, most likely CD8^+^ T-cells, are increased in COPD patients and CD8^+^ T-cells were shown to be involved in the development of CS-induced emphysema (83, 84). In addition to the N5 subset, the strong expression of key pro-inflammatory cytokines by neutrophils in general, including IL-1 family members (*Il1f9, Il1b)* (50) and *Tnf*, suggest that they are a second important regulator of smoke-induced pulmonary inflammation and tissue damage. The latter is supported by previous studies demonstrating that TNFR and IL1R knockout mice show considerably increased protection against smoke-induced emphysema as compared to wildtype mice (22). Consequently, our study supports that early targeting of neutrophils and importantly MoAM may be an auspicious approach to avoid excessive smoke-induced inflammation and tissue remodeling in the lung.

There is a pressing need for therapeutic interventions that target the inflammatory process and dysfunction underlying COPD (20). Based on our data, we pursued an approach to reduce the inflammatory burden and associated tissue remodeling in lungs by pharmacological inhibition of IRAK4. We have shown that IRAK4 inhibition significantly reduced the recruitment of neutrophils, specifically the N5 subset, and MoAM as well as key MoAM- and neutrophil-associated inflammatory mediators such as CCL2/CCL7 and IL-1β/TNF-α that are required to establish and maintain (smoke-induced) pulmonary inflammation and tissue remodeling/emphysema (12, 22, 72). Hence, our study indicates that pharmacological IRAK4 inhibition disrupts the smoke-induced self-amplifying inflammatory loop which makes it also an attractive therapeutic concept for COPD. The latter is supported by the fact that IL-1 is increased in COPD and involved in its pathogenesis and recent studies that suggest an important role of TLRs for initiating the inflammatory process and its development in COPD (10, 12, 85). In fact, we hypothesize that IRAK4 inhibition might be most appropriate in early disease, a strategy that is supported by recent evidence demonstrating that around 40% of terminal bronchioles are already lost in patients with mild COPD (86). We note that these are first promising proof-of-principal data that need to be confirmed in future studies, importantly these studies need to assess the impact of IRAK4 inhibitor treatment on pathological tissue remodeling and damage, e.g. using a chronic smoke model, as well as susceptibility to microbial infections. These future studies should also carefully examine the role of MoAM in small airway fibrosis since our data suggest functional similarity of the MoAM identified here and the MoAM identified as drivers of pulmonary fibrosis in a previous study based on their molecular profile (19). This is exemplified by the expression of the fibrogenic marker *Pdgfa* by MoAM.

One limitation of our study is that we analyzed cell pools from different animals, which does not allow assessment of the variability across animals. We note that equal cell counts were pooled per animal which excludes the possibility that the results are driven by single animals. The data sets of the acute, sub-chronic and chronic phase of smoke-induced lung inflammation were generated independently of each other using two different scRNA-seq platforms (10x Chromium and Drop-seq). However, our integrated analysis demonstrates that the same cell populations and even subpopulations were captured using both technologies, which strongly argues for only a minor bias if at all due to the different techniques. The consistency observed across these different data sets makes our findings even stronger and illustrate the potential of such integrative analyses to identify cellular and molecular alterations in the context of disease relevant processes. As there has recently been increasing interest in inflammatory COPD phenotypes, namely eosinophilic versus neutrophilic COPD, we want to note that eosinophils were not detected in our preclinical smoke model which is in line with previous findings (87, 88) and may limit the translatability of our findings to eosinophilic COPD. Whether this observation is related to the model or droplet-based scRNA-seq technology requires further investigation.

Overall, we have demonstrated that smoking provokes an excessive recruitment and accumulation of pro-inflammatory and tissue remodeling associated MoAM in the airspace. These recruited MoAM are together with neutrophils key drivers and amplifiers of the CS-induced inflammatory response in the lung. The massive MoAM increase in emphysema, which also translates to their human orthologs in emphysematous COPD patients, provides further evidence for their direct involvement in CS-induced lung pathology in humans and a major advancement in the understanding of the functional consequence of monocyte recruitment into the lung in response to smoking. Finally, our findings may lay the foundation for a better molecular stratification of COPD sub-populations and the development of tailored treatment strategies for them. In particular, this study provides a rationale for targeting MoAM and neutrophil subsets as well as their inflammatory signaling pathways in future clinical trials for COPD.

## Data availability statement

The datasets presented in this study can be found in online repositories. The names of the accession numbers and repositories and can be found below: PRJEB47060 (ENA; https://www.ebi.ac.uk/ena/browser/view/PRJEB47060) and EGAS00001004369 (EGA; https://ega-archive.org/studies/EGAS00001004369).

## Ethics statement

The studies involving humans were approved by Ethics committees of the University of Bonn and University hospital Bonn. The studies were conducted in accordance with the local legislation and institutional requirements. The participants provided their written informed consent to participate in this study. The animal study was approved by Boehringer Ingelheim animal welfare committee & ethical committee of the local authorities (Regierungspräsidium Tübingen 16-030-G, 35/9185.81-8). The study was conducted in accordance with the local legislation and institutional requirements.

## Author contributions

CW: Conceptualization, Data curation, Formal analysis, Investigation, Visualization, Writing – original draft, Writing – review & editing. KB: Investigation, Visualization, Writing – review & editing. CWa: Investigation, Writing – review & editing. YS: Data curation, Formal analysis, Writing – review & editing. GL: Formal analysis, Writing – review & editing. CT: Resources, Writing – review & editing. FH: Investigation, Writing – review & editing. DK: Investigation, Writing – review & editing. BS: Resources, Writing – review & editing. DD: Writing – review & editing. TB: Supervision, Writing – review & editing. FG: Resources, Supervision, Writing – review & editing. JS: Supervision, Writing – review & editing. CV: Conceptualization, Resources, Supervision, Writing – original draft. PB: Conceptualization, Resources, Supervision, Writing – original draft.
