# Supplementary material for: Monocyte-derived alveolar macrophages are key drivers of smoke-induced lung inflammation and tissue remodeling
Source: Front Immunol. 2024 Jan 29;15:1325090. doi: 10.3389/fimmu.2024.1325090 (PMC10859862; doi:10.3389/fimmu.2024.1325090)
Supplement: Supplementary file 1 [file DataSheet_1.pdf]

Supplementary Material

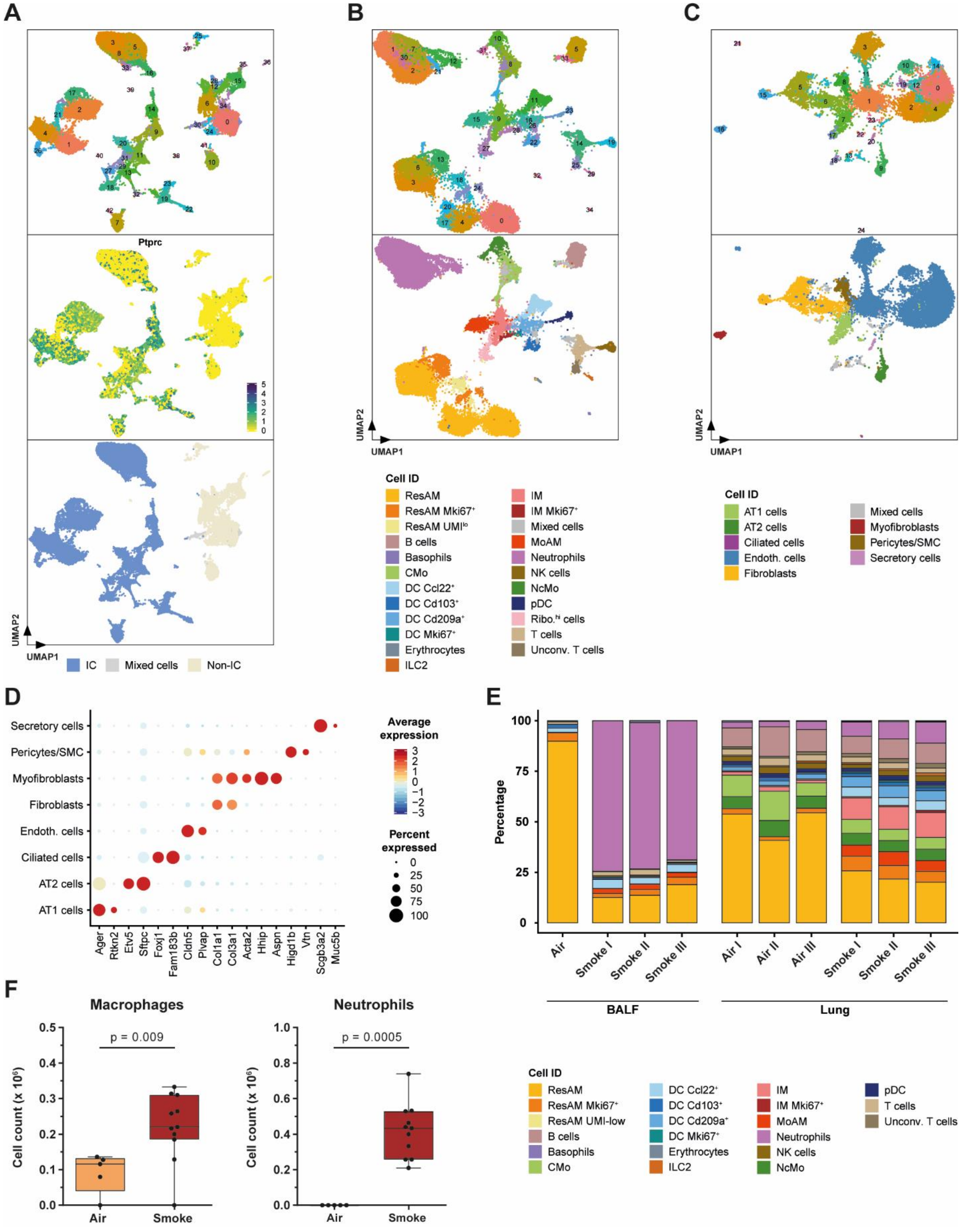

Supplementary Figure S1. Acute smoke model.

(A) UMAP plots represent computationally determined cell clusters (top panel) in the integrated data set of the acute smoke model, *Ptprc* (CD45) expression per single cell (middle panel) and classification of cell clusters into IC, non-IC and mixed cells that represent IC/non-IC multiplets (bottom panel). Classification was based on *Ptprc* expression and the gene panels shown in (D) and Fig. 2C. **(B, C)** UMAP plots illustrating computationally determined cell clusters (top panel) and annotated cell types (bottom panel) of the re-clustered IC (B) and non-IC (C). **(D)** Dot plot illustrating average scaled expression levels of markers used to annotate non-IC populations. Dot size represents the fraction of cells per cell population that express the respective marker gene. **(E)** Relative frequency of the different immune cell populations in lung tissue and BALF of smoke-exposed and air control animals. Data are shown per biological replicate. **(F)** Total macrophage and neutrophil counts in BALF of air control (n = 5) and smoke-exposed animals (n = 11) as determined by fluorescence flow cytometry. Statistical significance was determined by Mann-Whitney U test.

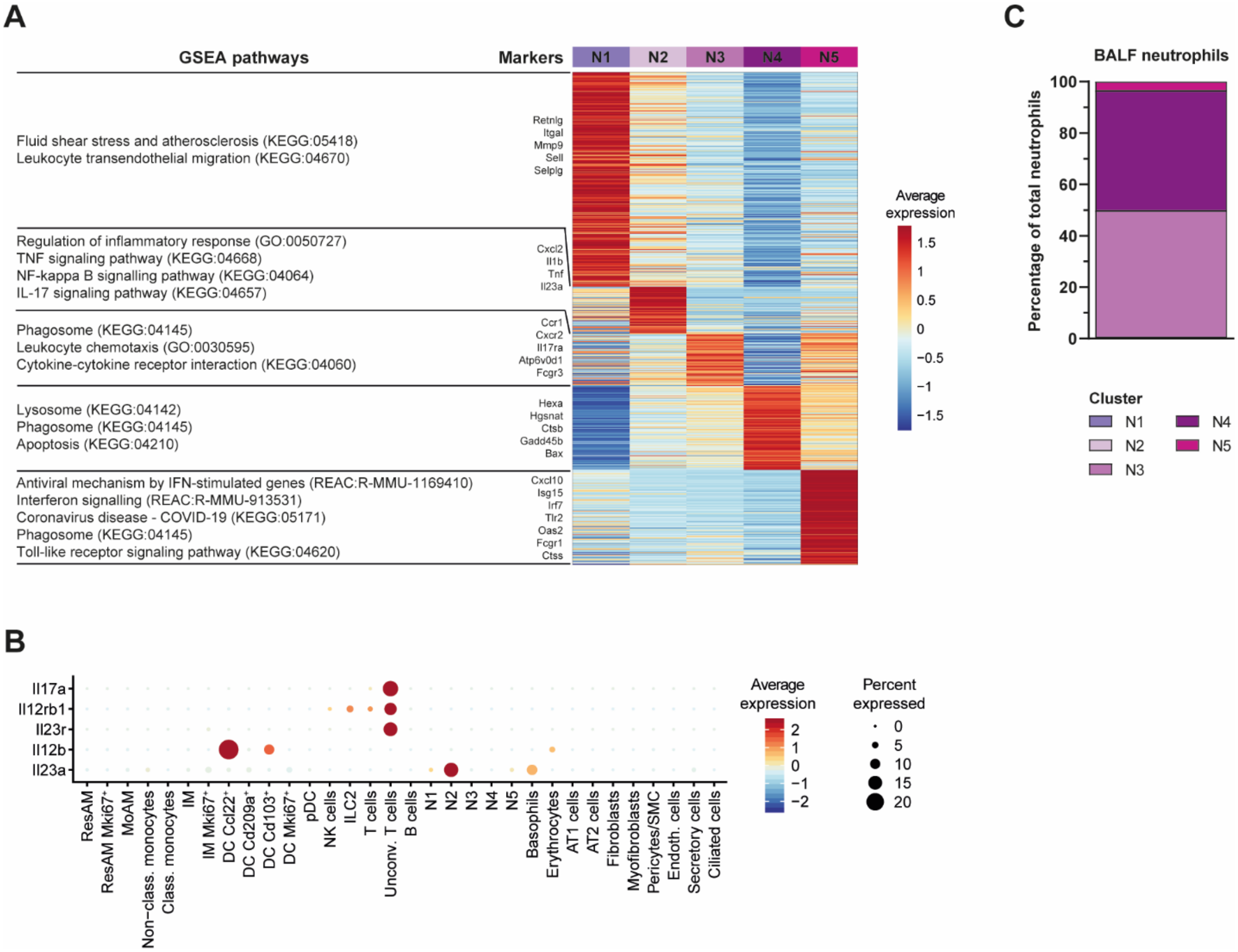

Supplementary Figure S2. Characterization of neutrophils.

(A) Heatmap representing the relative average expression of genes that are significantly differentially expressed (FC ≥ 1.5, adj. P < 0.05) across neutrophil subsets from smoke-exposed animals. Select molecular pathways significantly (adj. $P < 0.05$) associated with the respective subpopulation as determined by GSEA and marker genes are listed. **(B)** Dot plot illustrating relative average expression levels of genes encoding for IL-23 (*Il23a*, *Il12b*), IL-23R (*Il23ra*, *Il12rb1*) and IL-17A (*Il17a*) across the cell populations identified in smoke-exposed mice and the fraction of cells expressing the markers per cell population. **(C)** Relative frequency of neutrophil subsets in BALF of smoke-exposed animals.

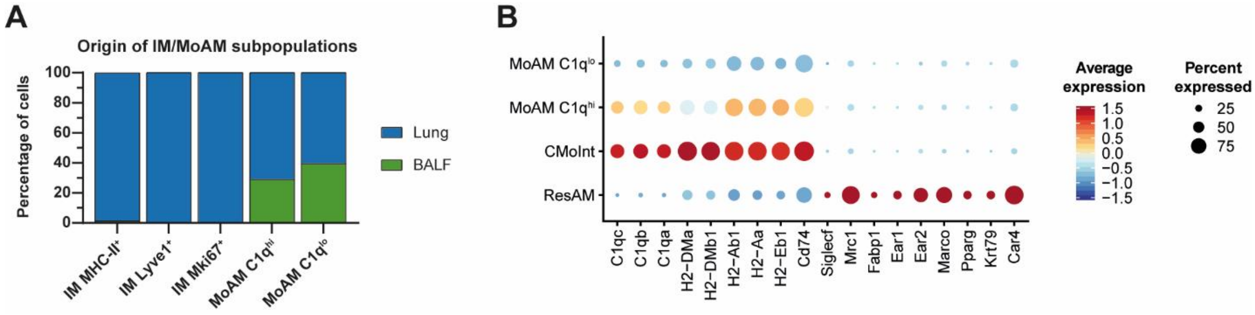

Supplementary Figure S3. Both MoAM subsets are present in BALF and do not express known ResAM markers.

(A) Bar plot illustrating the percentage of cells from IM and MoAM subsets of the integrated data set that derived from BALF and whole lung samples. **(B)** Dot plot illustrating average scaled expression levels of MoAM/CMoInt (C1q and MHC-II components) and ResAM marker genes across these cell populations and the fraction of cells that express the respective marker. Data are shown for cells isolated from smoke-exposed mice.

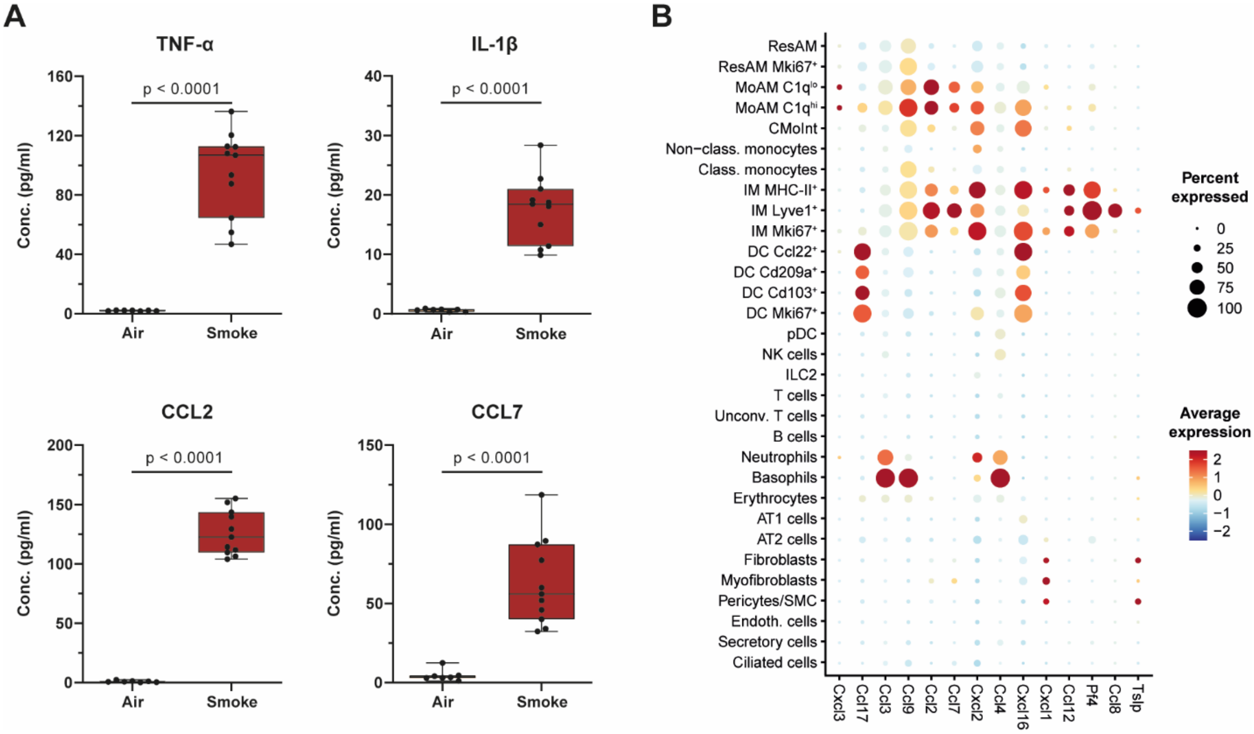

Supplementary Figure S4. Pro-inflammatory markers are induced in the murine lung by acute smoke exposure.

(A) TNF-α, IL-1β, CCL2 and CCL7 levels in BALF of air control (n = 7) and smoke-exposed mice (n = 11). Statistical significance was determined by Mann-Whitney U test. **(B)** Dot plot illustrating average scaled expression levels and the fraction of cells expressing MoAM and IM associated chemokines and *Tslp* across the cell populations and macrophage subpopulations identified in smoke-exposed mice.

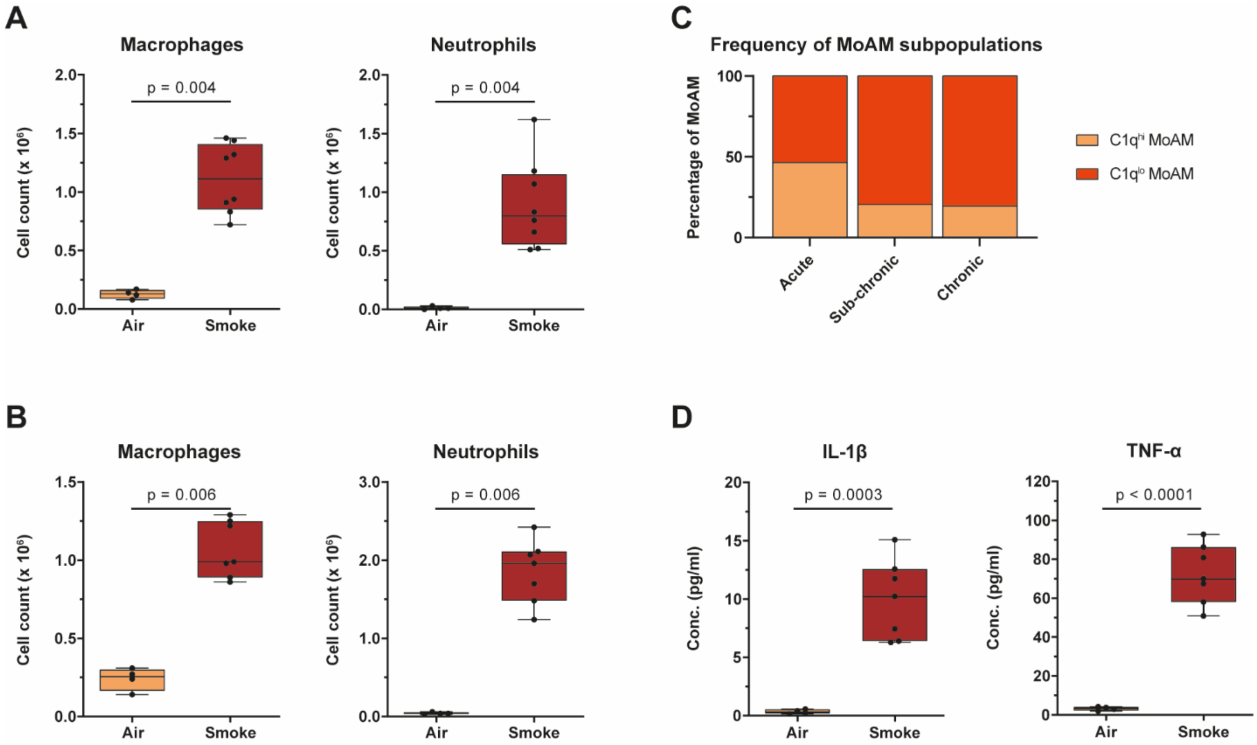

Supplementary Figure S5. Sub-chronic and chronic smoke models.

(A, B) Total macrophage and neutrophil counts in BALF of air control (n = 4) and smoke-exposed animals (sub-chronic: n = 8, chronic: n = 7) of the sub-chronic (A) and chronic (B) models as determined by fluorescence flow cytometry. Statistical significance was determined by Mann-Whitney U test. **(C)** Frequency of C1q^{hi} and C1q^{lo} MoAM subsets in BALF after acute, sub-chronic and chronic smoke exposure. **(D)** TNF-α and IL-1β levels in BALF of air control (n = 4) and chronically smoke-exposed mice (n = 7). Statistical significance was determined by Welch's t-test.

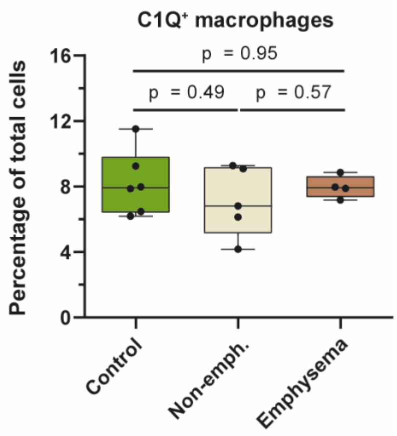

Supplementary Figure S6. Frequency of human C1Q$^+$ macrophages.

Relative frequency of C1Q$^+$ macrophages in control subjects (n = 6), non-emphysematous (n = 5) and emphysematous COPD/CPFE (n = 4) patients. Statistical significance was determined by Kruskal-Wallis/Dunn's multiple comparison test.

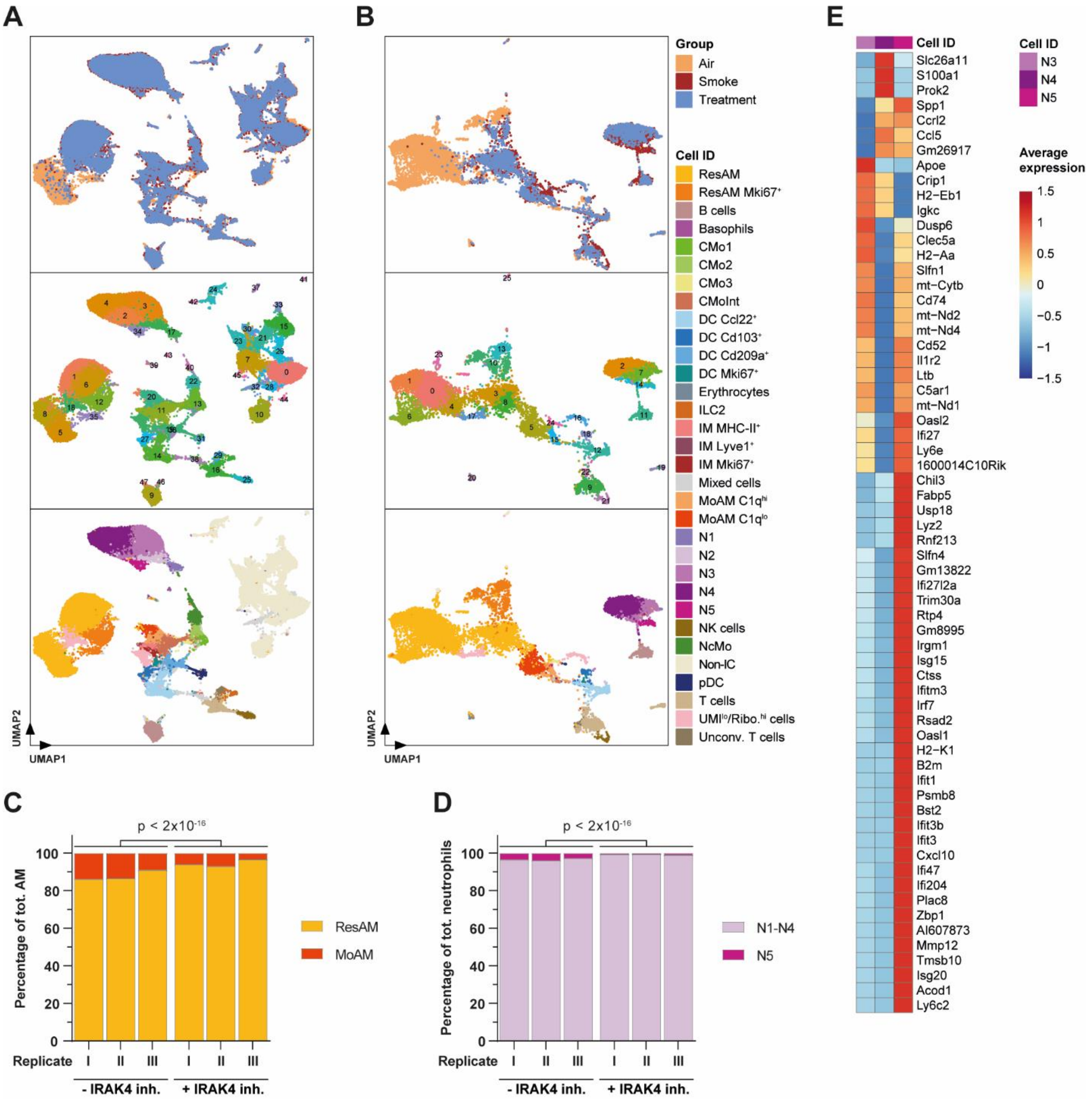

Supplementary Figure S7. IRAK4 inhibitor treatment.

(A, B) UMAP plots represent the integrated data sets of air control, smoke-exposed and treated mice after acute (A) and sub-chronic (B) smoke exposure. Cells are colored by experimental group (upper panel), computationally determined cell clusters (middle panel) and annotated cell types (bottom panels). **(C, D)** Percentage of MoAM and ResAM of total AM and the N5 subset of the total neutrophils in BALF per biological replicate of acute smoke-exposed control (-IRAK4 inh.) and treated (+ IRAK4 inh.) animals (n = 3-4 mice per replicate). Statistical significance was determined by a generalized linear model with binomial distribution on the absolute cell counts as determined by scRNA-seq (Supplementary Table S5 and Supplementary Table S6). **(E)** Relative expression of genes significantly down-regulated (FC ≥ 1.5, adj. $P < 0.05$) by treatment in the total BALF neutrophil population after sub-chronic smoke exposure across the major BALF neutrophil subsets.

Supplementary Table S1. Summary of cells analyzed per sample.

Model	Group	Replicate	BALF cell count	Lung cell count
**Acute**	Air	1	3,084 (3,041)	4,298 (3,281)
		2	N/A (N/A)	4,295 (2,697)
		3	N/A (N/A)	3,974 (2,210)
	Smoke	1	3,201 (3,195)	4,759 (3,140)
		2	3,879 (3,851)	5,217 (2,785)
		3	3,173 (3,162)	5,131 (2,652)
	Treatment	1	3,522 (3,477)	5,122 (2,571)
		2	4,034 (3,988)	5,254 (2,496)
		3	3,907 (3,873)	4, 539 (2,511)
**Sub-chronic**	Air	1	5,420 (5,376)	N/A
	Smoke	1	2,352 (2,341)	N/A
	Treatment	1	2,455 (2,439)	N/A
**Chronic**	Air	1	108 (96)	N/A
	Smoke	1	990 (987)	N/A

Numbers in brackets specify the number of immune cells.

Supplementary Table S2. Contingency table for statistical analysis of the acute smoke effect on proliferation of ResAM.

Compartment	Group	ResAM count	ResAM Mki67$^+$ count
BALF	Air	2,731	127
	Smoke	1,526	283
Whole lung	Air	4,066	187
	Smoke	1,941	557

Supplementary Table S3. Contingency table for statistical analysis of the smoke effect on ResAM and MoAM frequency.

Model	Group	ResAM count	MoAM count
Acute	-IRAK4 inh.	1,814	246
	+IRAK4 inh.	3,891	230
Sub-chronic	-IRAK4 inh.	674	302
	+IRAK4 inh.	927	257

Total ResAM and MoAM collected from BALF of smoke-exposed controls (-IRAK4 inh.) and smoke-exposed treated (+IRAK4 inh.) animals after acute and sub-chronic smoke exposure.

Supplementary Table S4. Contingency table for statistical analysis of the smoke effect on neutrophil subset N5 frequency.

Model	Group	N1-4 count	N5 count
Acute	-IRAK4 inh.	7,087	259
	+IRAK4 inh.	6,113	52
Sub-chronic	-IRAK4 inh.	622	82
	+IRAK4 inh.	670	2

Total N1-4 neutrophils and N5 neutrophils collected from BALF of smoke-exposed controls (-IRAK4 inh.) and smoke-exposed treated (+IRAK4 inh.) animals after acute and sub-chronic smoke exposure.

Supplementary Table S5. Contingency table for statistical analysis of the acute smoke effect on neutrophil subset N5 frequency across biological replicates.

Group	Replicate	N1-N4 count	N5 count
-IRAK4 inh.	1	2,294	86
	2	2,677	112
	3	2,116	61
+IRAK4 inh.	1	1,606	13
	2	2,353	16
	3	2,154	23

Total N1-4 neutrophils and N5 neutrophils collected from BALF of smoke-exposed controls (-IRAK4 inh.) and smoke-exposed treated (+IRAK4 inh.) animals after acute smoke exposure. Data are provided per biological replicate.

Supplementary Table S6. Contingency table for statistical analysis of the acute smoke effect on ResAM and MoAM frequency across biological replicates.

Group	Replicate	ResAM count	MoAM count
-IRAK4 inh.	1	469	76
	2	636	100
	3	709	70
+IRAK4 inh.	1	1,426	93
	2	1,168	90
	3	1,297	47

Total ResAM and MoAM collected from BALF of smoke-exposed controls (-IRAK4 inh.) and smoke-exposed treated (+IRAK4 inh.) animals after acute smoke exposure. Data are provided per biological replicate.
